# When the Liver Echoes to the Heart: Assessing Subclinical Cardiac Dysfunction in NAFLD Using Speckle Tracking Echocardiography—A Systematic Review and Meta-Analysis

**DOI:** 10.3390/biomedicines13122908

**Published:** 2025-11-27

**Authors:** Micha Gruber, Malaz Almasri, Rania Abdulredha, Iulia Tecar, Daniel-Corneliu Leucuta, Stefan-Lucian Popa, Dan L. Dumitrascu, Abdulrahman Ismaiel

**Affiliations:** 1 Faculty of Medicine, “Iuliu Hatieganu” University of Medicine and Pharmacy, 400006 Cluj-Napoca, Romania; micha.h.gruber@gmx.de (M.G.); rania_live@hotmail.de (R.A.); 2 Dr. Constantin Papilian Cluj-Napoca Emergency Military Hospital, “Iuliu Hatieganu“ University of Medicine and Pharmacy, 400347 Cluj-Napoca, Romania; malathalmasri@yahoo.com; 3 Department of Cardiology, “Nicolae Stăncioiu” Heart Institute, 400001 Cluj-Napoca, Romania; iulia.tecar@yahoo.com; 4 Department of Medical Informatics and Biostatistics, “Iuliu Hatieganu” University of Medicine and Pharmacy, 400349 Cluj-Napoca, Romania; 5 2nd Department of Internal Medicine, “Iuliu Hatieganu” University of Medicine and Pharmacy, 400006 Cluj-Napoca, Romania; popa.stefan@umfcluj.ro (S.-L.P.); ddumitrascu@umfcluj.ro (D.L.D.); abdulrahman.ismaiel@yahoo.com (A.I.)

**Keywords:** non-alcoholic fatty liver disease (NAFLD), metabolic-dysfunction-associated steatotic liver disease (MASLD), heart failure (HF), subclinical left ventricular systolic dysfunction, myocardial strain, speckle tracking echocardiography

## Abstract

**Introduction:** Worldwide, non-alcoholic fatty liver disease (NAFLD) is the most prevalent chronic liver disorder, strongly associated with increased cardiovascular morbidity and mortality. Although patients have a preserved left ventricular ejection fraction (LVEF), individuals having NAFLD may demonstrate subclinical cardiac dysfunction. Speckle tracking echocardiography (STE) enables a more sensitive evaluation, identifying even subtle alterations of myocardial strain, compared to conventional LVEF measurements. This systematic review and meta-analysis sought to examine the relationship between NAFLD and subclinical left ventricular systolic impairment, utilizing STE-derived strain parameters. **Methods:** A comprehensive search of the literature was undertaken using PubMed, EMBASE, and Scopus. Observational studies evaluating patients with NAFLD through STE-derived myocardial strain parameters were included. Study quality was appraised using the Newcastle-Ottawa Scale. The primary outcomes were the mean differences (MD) in global longitudinal strain (GLS), global circumferential strain (GCS), global radial strain (GRS), global area strain (GAS), and related strain rate indices between NAFLD spectrum patients and controls. **Results:** A total of sixteen studies, comprising 8359 participants, were included in the analysis. Compared to controls, patients with NAFLD demonstrated significant reductions in GLS (MD: −2.043; 95% CI: −2.868, −1.218), GAS (MD: −3.706; 95% CI: −4.999, −2.413), and GCS (MD: −1.415; 95% CI: −2.893, 0.064). These reductions were more substantial among individuals with moderate to severe NAFLD and those with concomitant type 2 diabetes mellitus (GLS MD: −4.385; 95% CI: −5.400, −3.369 in diabetic NAFLD vs. diabetic controls). Subgroup analysis further revealed a progressive deterioration in strain parameters from simple steatosis to more severe NAFLD. Notably, LVEF remained preserved in all groups, highlighting the subclinical nature of this dysfunction. **Conclusions:** This meta-analysis verifies the presence of subclinical left ventricular systolic dysfunction in individuals with NAFLD, which is identifiable by STE despite preserved LVEF. Myocardial strain metrics, particularly GLS, serve as sensitive early markers of myocardial impairment. Routine application of STE in the clinical assessment of NAFLD may support earlier cardiovascular risk detection and timely intervention.

## 1. Introduction

Non-alcoholic fatty liver disease (NAFLD) has emerged as the leading cause of chronic liver disease globally. NAFLD is characterized by the accumulation of hepatic fat in the absence of significant alcohol consumption or other secondary etiologies [1]. The disease is associated with a substantial increase in both morbidity and mortality, as evidenced by a wide range of intrahepatic and extrahepatic manifestations [2]. Most significantly, numerous studies have established a strong association between NAFLD and cardiovascular complications [3]. In fact, it is the primary cause of mortality in patients with NAFLD [4]. It is often linked to cardiovascular disease, driven by both structural and functional alterations in the heart [5]. Structural modifications associated with NAFLD include left ventricular hypertrophy, increased epicardial fat thickness, and valvular calcification [6]. Functional alterations, on the other hand, involve diastolic dysfunction, conduction abnormalities, prolonged QTc intervals, and cardiac arrhythmias, including those of both atrial and ventricular origin [7].

In addition to overt cardiovascular complications, NAFLD has been linked to subclinical myocardial dysfunction [8], which has significant implications in the pathophysiology of several related conditions. Early detection and management of subclinical myocardial dysfunction are therefore of paramount importance to mitigate the progression of cardiovascular disease in this patient population. Over the years, several echocardiographic parameters have been developed for the non-invasive assessment of left ventricular (LV) systolic function. Among these, left ventricular ejection fraction (LVEF) remains the most widely evaluated parameter. However, LVEF is not a direct measurement of systolic function but rather an estimation of myocardial contractile function. This reliance on estimation introduces certain limitations [9]. LVEF can be influenced by variables such as heart rate and loading conditions, and more importantly, it is unable to detect subtle alterations in contractile function, as discussed previously [10]. Given these limitations, it becomes clear that an alternative method is required to accurately assess subclinical myocardial damage, which may otherwise contribute to the development of additional pathologies [11].

In response to the need for more accurate methods, other ultrasound-based techniques have been introduced. One such technique is speckle tracking echocardiography (STE), a non-invasive imaging method that enables the quantitative assessment of both global and regional myocardial function [10,12]. STE has the advantage of being independent of insonation angle and cardiac translational movements, thus offering a more precise evaluation of myocardial strain. Strain is quantified by measuring the percentage change in the length of a myocardial segment over a specified period [12,13]. By utilizing STE, it is possible to overcome the limitations inherent to LVEF, thereby facilitating the detection of early LV dysfunction, even in individuals with preserved ejection fraction [14]. STE is a sophisticated imaging technique that utilizes natural speckles within the myocardium to quantify myocardial deformation. This method allows for the assessment of the LV systolic function, for example, global longitudinal strain (GLS), global circumferential strain (GCS), global radial strain (GRS), global area strain (GAS), and several other indices. GLS measures the percentage of myocardial shortening along the long axis of the heart, providing a sensitive indicator of left ventricular systolic function. GAS, global area strain, quantifies the percentage change in myocardial area during the cardiac cycle, combining the effects of longitudinal and circumferential deformation, while GCS quantifies circumferential myocardial deformation. GRS quantifies the degree of myocardial wall thickening during systole, reflecting the radial contractile function of the left ventricle. These and various other STE parameters offer superior sensitivity to traditional ejection fraction measurements, enabling early detection of subtle cardiac dysfunction and potentially guiding more precise clinical management strategies [15].

While earlier studies suggested no association between NAFLD and reductions in LVEF [14,16,17,18], more recent research using advanced techniques such as STE has demonstrated a link between NAFLD and subclinical LV systolic dysfunction [19]. However, these studies are often single-center and include varied populations, creating a clinical gap where the precise magnitude and consistency of this dysfunction remain unclear. Therefore, the rationale for this study was to synthesize the available evidence to quantify this impairment. We hypothesized that NAFLD is associated with significant subclinical left ventricular systolic dysfunction, detectable by STE-derived strain parameters (particularly GLS), despite a preserved LVEF. We further hypothesized that this myocardial impairment would be more pronounced in patients with more severe stages of NAFLD and in those with concomitant type 2 diabetes [20]. Given these hypotheses, we decided to conduct this systematic review and meta-analysis to systematically evaluate subclinical LV systolic dysfunction in patients with NAFLD, specifically using myocardial strain measurements obtained through STE.

## 2. Methods

We wrote our systematic review and meta-analysis according to the Preferred Reporting Items for Systematic Reviews and Meta-Analyses (PRISMA) checklist 2020 [21]. The study protocol has been registered in protocols.io (DOI: 10.17504/protocols.io.dm6gpmqypgzp/v1).

### 2.1. Data Sources and Search Strategy

Our goal was to review current evidence published on three databases: EMBASE, PubMed, and Scopus, including observational studies evaluating the association between NAFLD and subclinical LV systolic dysfunction measured by myocardial strain STE. We included all articles available in full text published until the 28 October 2023. A detailed explanation of the search strategy employed in this study is available in the Supplementary Material S1. This includes the databases queried, the specific search terms, and the keywords used. No restrictions were applied based on study duration, language, or country of origin in the search strategy. Although all languages were considered in the initial search, only full texts meeting our inclusion criteria were reviewed. Our comprehensive search process began with the screening of titles and abstracts. After this initial screening, studies that met the predefined inclusion criteria were subjected to qualitative synthesis, and selected articles underwent full-text review. In addition, eligibility criteria were rigorously applied to all evaluated studies, followed by data extraction. Two independent authors (G.M. and I.T.) performed the data extraction process separately; any discrepancies between the extractions were resolved through a second evaluation and mutual consensus. The following data were extracted from each study: study name, country of origin, year of publication, study type, total number of subjects, percentage of subjects with NAFLD, mean age, body mass index (BMI), method of NAFLD diagnosis, gender distribution, LVEF, available strain parameters, and a summary of the study’s conclusions.

### 2.2. Eligibility Criteria

The inclusion criteria for original articles were as follows: (1) Full-text studies employing observational cohort, population-based or hospital-based, cross-sectional, or case–control designs that assessed subclinical LV systolic dysfunction through myocardial strain analysis using STE; (2) NAFLD diagnosis confirmed by hepatic steatosis, as determined by liver biopsy or imaging modalities such as ultrasonography, computed tomography (CT), or magnetic resonance imaging (MRI), with exclusion of secondary causes of hepatic steatosis and significant alcohol intake; (3) Absence of other chronic liver diseases (CLD) or liver cirrhosis; (4) Adult participants aged 18 years or older, without restrictions on gender, race, or ethnicity; (5) Human studies exclusively; and (6) Articles published in English, German, or Romanian.

The exclusion criteria included: (1) Studies involving secondary causes of hepatic steatosis or significant alcohol intake; (2) Presence of any type of hepatitis virus infection; (3) Other known causes of chronic liver disease; (4) Diagnosed liver cirrhosis of any etiology; (5) Patients with end-stage liver disease on the liver transplantation waiting list; and (6) Editorials, letters to the editor, case reports, conference abstracts, literature reviews, systematic reviews, practice guidelines, commentaries, or abstracts without full-text availability.

### 2.3. Risk of Bias Assessment in Individual Studies

The risk of bias in individual studies was assessed using the quality assessment tool Newcastle-Ottawa Scale (NOS) [22], which is used to evaluate the quality of non-randomized studies in meta-analysis. We employed a single instrument for the assessment of cross-sectional studies. Each study’s internal validity and bias risk were evaluated using this instrument. Two writers (G.M. and R.A.) separately carried out the evaluations. In case of disagreement, a consensus was reached through a discussion.

### 2.4. Summary Measures and Synthesis of Results

The primary outcome of this analysis was the mean difference (MD) in various strain parameters evaluated using STE. Meta-analysis was performed using R with Metafor package (OpenMeta [Analyst]) [23]. The χ^2^-based Q-test and I^2^ were used to evaluate between-study heterogeneity. The random-effects model, along with MD, was utilized to estimate the overall effect size. For studies reporting medians with interquartile ranges or ranges, the mean and standard deviation (SD) were calculated. In studies with multiple subgroups of NAFLD patients or control subjects, group data were combined according to guidelines from the Cochrane Handbook. Subgroup analyses were conducted based on the presence of simple steatosis, moderate to severe steatosis, non-alcoholic steatohepatitis (NASH, both borderline and definitive), specific grades of liver fibrosis, the presence of type 1 or type 2 diabetes mellitus (T2DM), and gender differences, as provided by the extracted data. The results were presented as estimated MDs with 95% confidence intervals (CI), and statistical significance was determined by a *p*-value of <0.05.

## 3. Results

### 3.1. General Results

**Figure 1:**
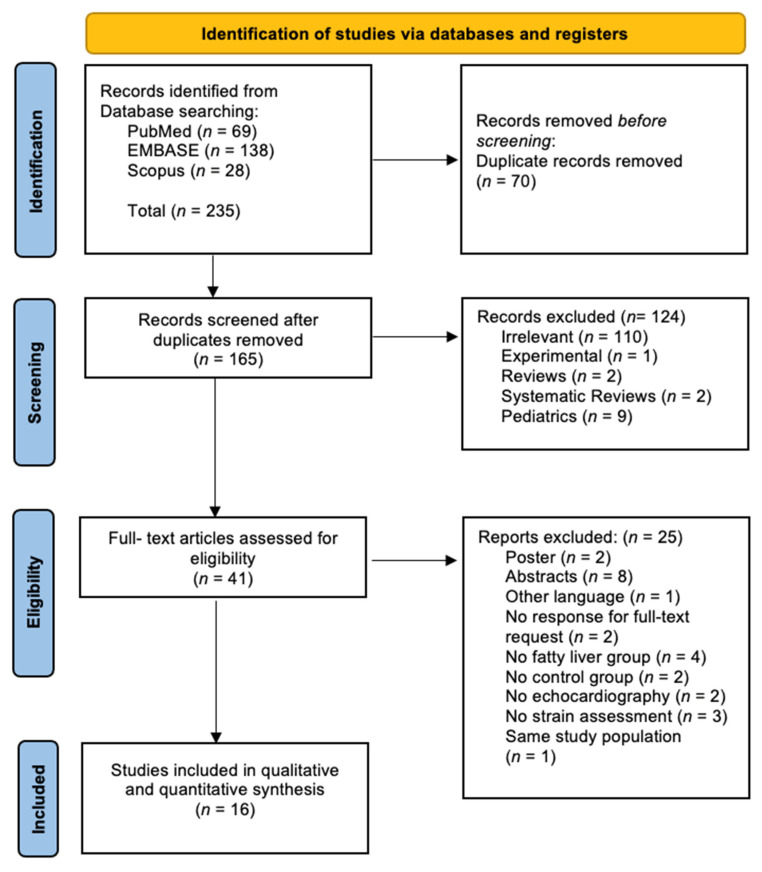
Summary of the identification, screening, and inclusion phases of our systematic review and meta-analysis according to the PRISMA flow diagram.

The initial search identified a total of 235 articles (PubMed: 69 articles, EMBASE: 138 articles, Scopus: 28 articles), as illustrated in Figure 1. Seventy studies were identified as duplicates and subsequently removed. Following this, 165 articles remained and were screened for eligibility based on inclusion and exclusion criteria through title and abstract review. The results of this screening were as follows: (1) 4 review articles (literature reviews: *n* = 2, systematic reviews: *n* = 2), (2) 9 pediatric studies, (3) 1 experimental study, and (4) 110 irrelevant studies. In total, 124 studies were excluded during this initial screening phase. The full texts of the remaining 41 articles were then thoroughly reviewed to assess eligibility for inclusion in the study. Of these, 25 were excluded for the following reasons: (1) 1 article was published in a language other than English, German, or Romanian (Chinese: 1 article) [24], (2) 8 articles were available only as abstracts, despite meeting the primary criteria [16,25,26,27,28,29,30,31], (3) 2 posters which met the primary criteria [32,33], (4) 2 articles missing a response to full-text requests [34,35], (5) 4 articles did not include a fatty liver group [36,37,38,39], (6) 2 articles where a healthy control group was absent [40,41], (7) 2 articles did not utilize echocardiography for cardiac assessment [5,42], (8) 3 articles used cardiac assessment methods other than strain [43,44,45], and (9) 1 article used the same population group as another study, already utilized for analysis [46]. Subsequently, a total of 41 articles were assessed for eligibility. Of these, 16 articles were deemed suitable for qualitative and quantitative synthesis [8,12,14,17,18,47,48,49,50,51,52,53,54,55,56,57].

### 3.2. Study Characteristics

A summary of the main characteristics of included studies is shown in Table 1 and Supplementary Table S1. This systematic review and meta-analysis included a total number of 8359 individuals. The sex distribution was higher for males (females—4109 [49%], males—4250 [51%]), while one study did not mention gender distribution [48]. NAFLD was present in 2327 subjects (27%) out of the total study sample, while NASH was present in 120 subjects (1.1%). Seven studies were undertaken in Europe (Italy *n* = 2, Turkey *n* = 3, Romania *n* = 2), three in the Middle East (Iran *n* = 2, Iraq *n* = 1), three studies in Asia (China *n* = 2, Taiwan *n* = 1), and three studies in the USA (*n* = 3).

### 3.3. Definition of Hepatic Steatosis

Hepatic steatosis and steatohepatitis were assessed using ultrasonography for diagnosing NAFLD in most studies (*n* = 9) [13,17,18,47,48,49,52,55,56], while the remaining studies used liver biopsy (*n* = 5) [8,50,53,54,57] and CT-scan (*n* = 2) [14,51]. Moreover, only one study used the MAFLD criteria [56].

### 3.4. Assessment of Strain Metrics and Additional Parameters

Additional metrics related to cardiac function were systematically analyzed, as outlined in Supplementary Figures S1–S13. Since LVEF is a globally recognized and widely accepted parameter, we decided to include it in the strain parameter section, although our primary focus was the evaluation of myocardial strain. In addition to our comprehensive analysis of the main STE parameters outlined in the introduction, we incorporated LVEF assessment, all of which are presented in Table 2 and STE Figure 2, Figure 3, Figure 4 and Figure 5.

### 3.5. Strain and LVEF Assessment in Controls vs. NAFLD

We evaluated the LVEF across 13 studies that compared control subjects to NAFLD patients [8,13,14,17,18,47,48,49,50,51,52,55,56], revealing an overall MD of 0.359 (95% CI −0.078, 0.795). Non-significant heterogeneity was observed, with an I^2^ = 26.74% and a *p*-value = 0.063. Leave-one sensitivity analysis was performed as reported in Supplementary Figure S1 and showed no significant difference.

GLS was assessed in 13 studies that compared control subjects to patients with NAFLD [8,13,14,17,18,47,48,49,50,51,52,55,56], with an overall MD of −2.043 (95% CI −2.868, −1.218). Considerable heterogeneity was observed, with an I^2^ = 96.98% and a *p*-value < 0.001. Leave-one sensitivity analysis was performed as demonstrated in Supplementary Figure S2 and showed a significant difference that remained.

The global strain rate (GSRS) parameter was examined across three studies that compared control subjects to NAFLD patients [49,50,52]. The combined analysis showed an overall MD of −0.033 (95% CI −0.078, 0.011). Heterogeneity was reported as not important with an I^2^ = 0.2% and *p*-value = 0.256.

Early diastolic strain rate (SRearly) was evaluated in a total of three studies comparing controls to NAFLD patients [49,50,52]. The pooled studies for the analysis demonstrated an overall MD of 0.072 (95% CI 0.015, 0.128). Insignificant heterogeneity was reported with an I^2^ = 0% and *p*-value = 0.411.

The late diastolic strain rate (SRlate) parameter was analyzed in three studies that compared a control group to individuals having NAFLD [49,50,52]. The overall analysis indicated an overall MD of 0.010 (95% CI −0.091, 0.111). Substantial heterogeneity was reported with an I^2^ = 73.54% and *p*-value = 0.025.

GCS was assessed in three studies that compared control subjects to NAFLD patients [13,51,55], revealing an overall MD of −1.415 (95% CI −2.893, 0.064). Considerable heterogeneity was reported with an I^2^ = 82.98% and *p*-value = 0.002.

GAS was assessed in two studies that compared a control group to an NAFLD group [13,55], indicating an overall MD of −3.706 (95% CI −4.999, −2.413). Insignificant heterogeneity was reported with an I^2^ = 0% and *p*-value = 0.461.

GRS was determined in two studies that compared a control group to individuals having NAFLD [13,55], demonstrating an overall MD of 7.407 (95% CI −1.852, 16.666). Considerable heterogeneity was reported with an I^2^ = 91.4% and *p*-value < 0.001.

### 3.6. Strain and LVEF Assessment in Controls vs. Simple Steatosis

We determined LVEF in three studies, which compared control subjects to patients with simple steatosis [13,50,55], revealing an overall MD of 0.300 (95% CI −1.529, 2.129). Non-significant heterogeneity was observed, with an I^2^ = 0.6% and a *p*-value = 0.416.

GLS was analyzed in three studies that compared control subjects to patients with simple steatosis [13,50,55], showing an overall MD of −2.253 (95% CI −3.502, −1.004). Moderate heterogeneity was reported with an I^2^ = 47.73% and *p*-value = 0.149.

GCS was evaluated in two studies that compared control subjects to patients with simple steatosis [13,55], revealing an overall MD of −0.851 (95% CI −2.575, 0.872). Moderate heterogeneity was reported with an I^2^ = 48.19% and *p*-value = 0.165.

GAS was assessed in two studies that compared control subjects to patients with simple steatosis [13,55], showing an overall MD of −1.383 (95% CI −2.944, 0.178). No relevant heterogeneity was reported with an I^2^ = 0% and *p*-value = 0.467.

GRS was assessed in two studies that compared control subjects to patients with simple steatosis [13,55], showing an overall MD of 2.128 (95% CI −1.927, 6.184). A non-significant heterogeneity was shown with an I^2^ = 29.92% and *p*-value = 0.232.

### 3.7. Strain and LVEF Assessment in Controls vs. Moderate/Severe NAFLD

We evaluated LVEF across two studies, which compared control subjects to patients with moderate to severe NAFLD [13,55], revealing an overall MD of 0.619 (95% CI −1.359, 2.596). Unimportant heterogeneity was observed, with an I^2^ = 9.99% and a *p*-value = 0.292.

GLS was assessed in controls vs. moderate/severe NAFLD patients with a total of two studies [13,55], with an overall MD of −5.828 (95% CI −7.496, −4.160). Substantial heterogeneity was reported with an I^2^ = 60.28% and *p*-value = 0.113.

GCS was investigated in two studies [13,55], indicating an overall MD of −3.111 (95% CI −4.630, −1.593). Heterogeneity was reported as potentially moderate with an I^2^ = 45.31% and *p*-value = 0.176.

GAS was assessed in two studies [13,55], showing an overall MD of −5.231 (95% CI −6.707, −3.756). Heterogeneity was defined as irrelevant with an I^2^ = 0% and *p*-value = 0.428.

GRS parameter was analyzed in two studies [13,55], demonstrating an overall MD of 9.936 (95% CI −0.141, 20.013). Significant heterogeneity with an I^2^ = 93.01% and *p*-value < 0.001 was reported.

### 3.8. Strain and LVEF Assessment in Simple Steatosis vs. Moderate/Severe NAFLD

We evaluated LVEF across 2 studies, which compared subjects with simple steatosis to patients with moderate to severe NAFLD [13,55], revealing an overall MD of 0.585 (95% CI −1.231, 2.402). Unimportant heterogeneity was observed, with an I^2^ = 0% and a *p*-value = 0.629.

GLS was analyzed in two studies [13,55], showing an overall MD of −3.684 (95% CI −4.956, −2.412). Non-significant heterogeneity was reported with an I^2^ = 0% and *p*-value = 0.847.

Two studies were included to evaluate GCS for comparing simple steatosis with moderate to severe NAFLD [13,55]. The combined analysis of studies demonstrated an overall MD of −2.126 (95% CI −3.335, −0.917). Unimportant heterogeneity was reported with an I^2^ = 0% and *p*-value = 0.862.

Two studies were analyzed for GAS [13,55], revealing an overall MD of −3.939 (95% CI −6.288, −1.591). Heterogeneity was reported as moderate with an I^2^ = 44.62% and *p*-value = 0.179.

**Figure 2:**
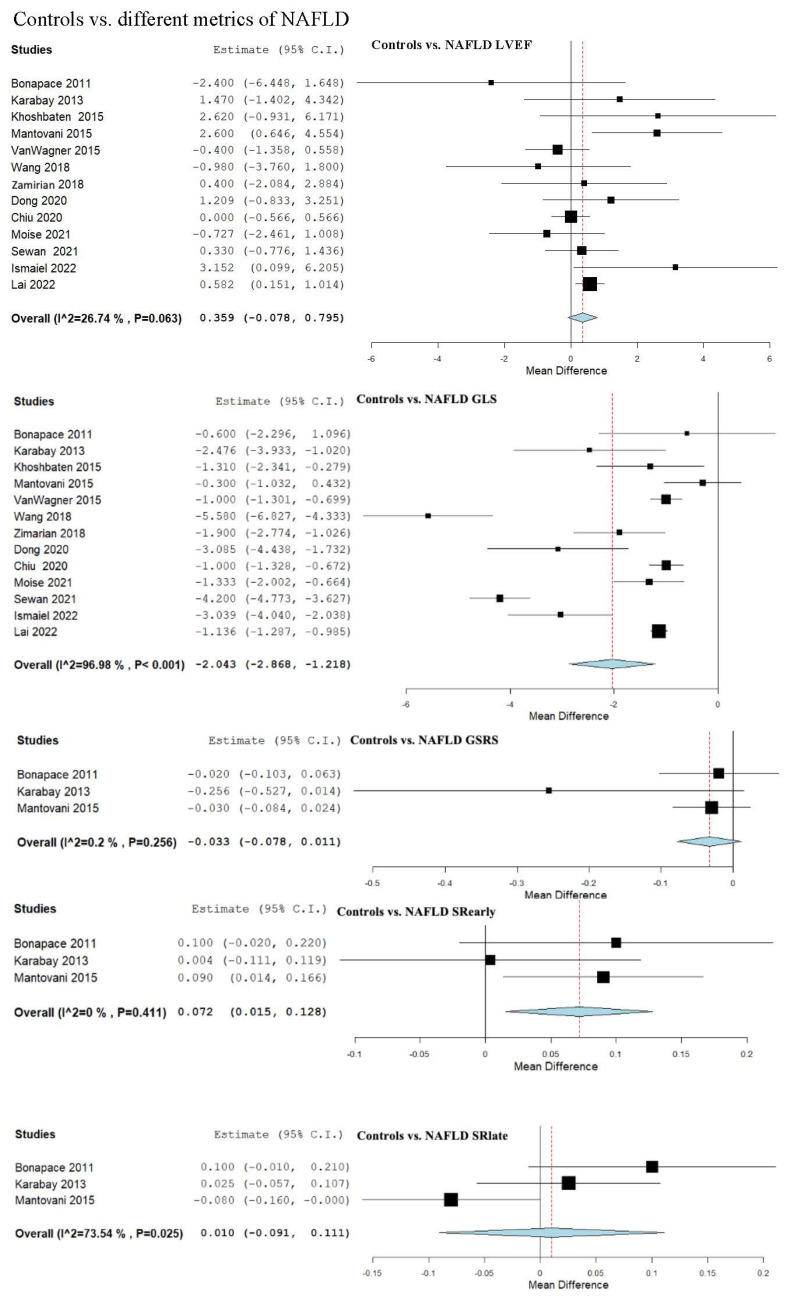

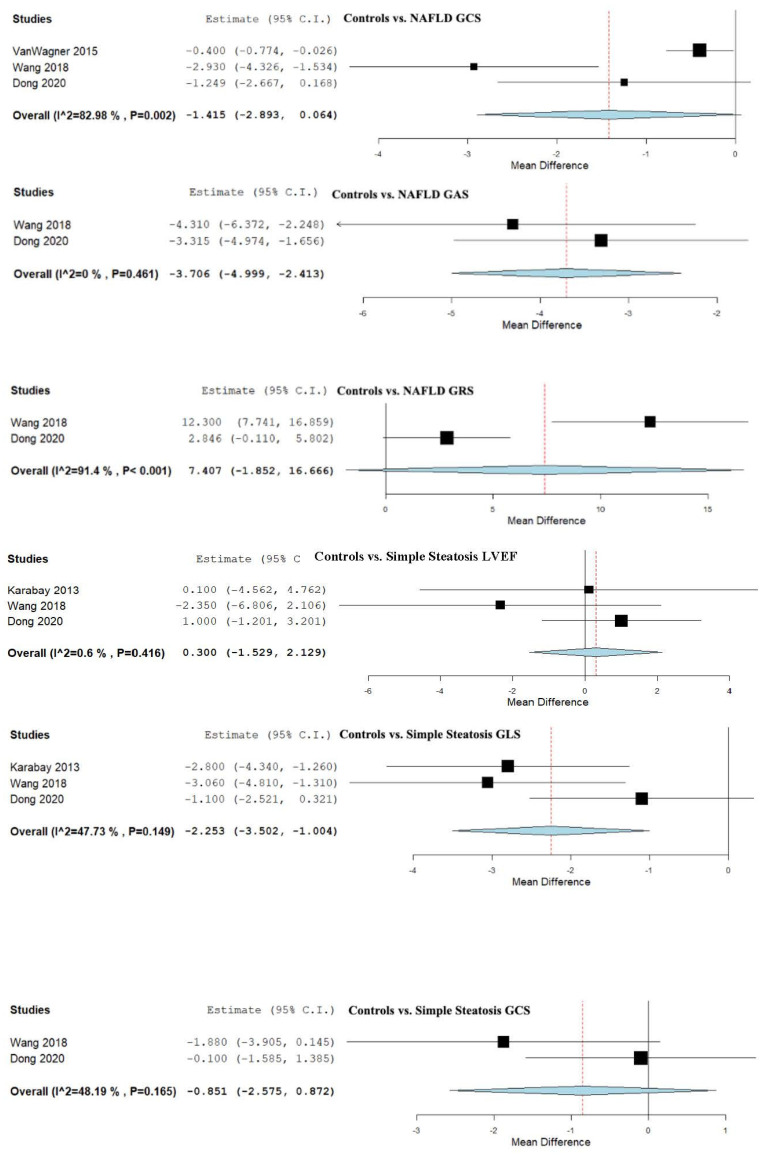

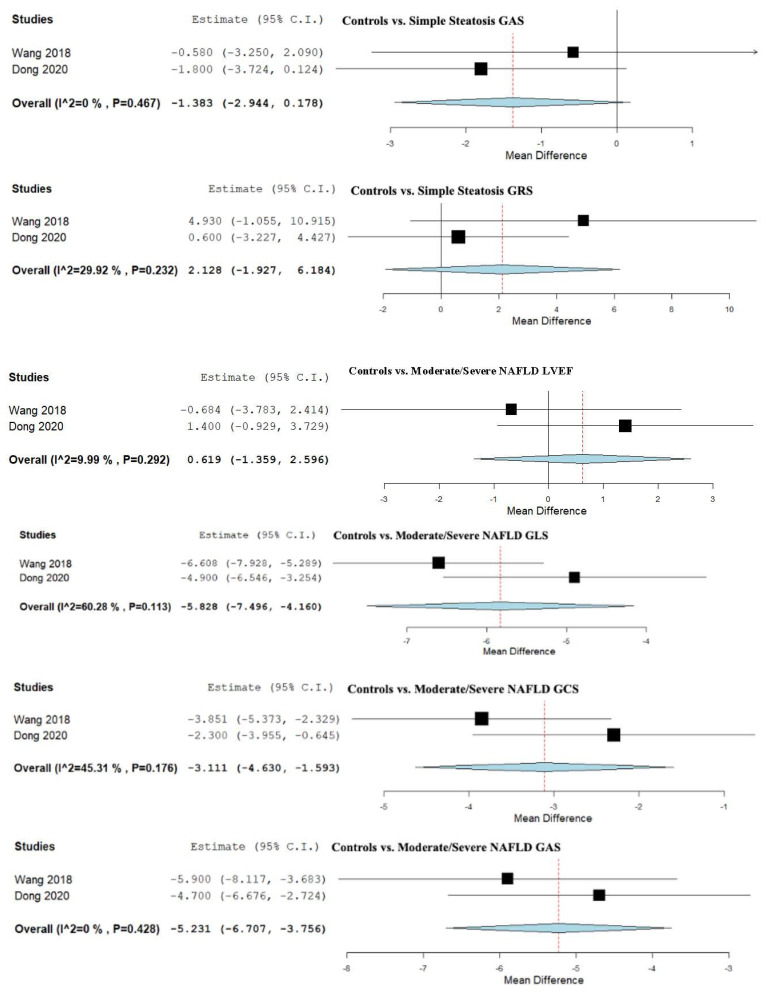

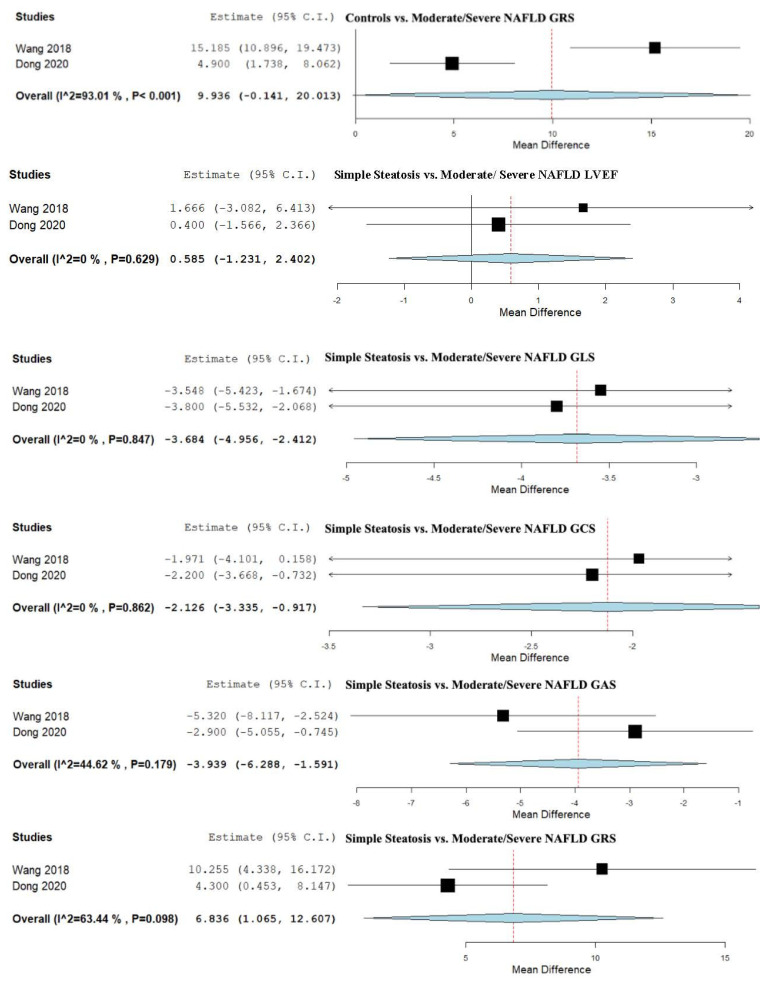
Studies evaluating myocardial strain parameters in Controls vs. NAFLD, Controls vs. Simple Steatosis, Controls vs. Moderate/Severe NAFLD, and Simple Steatosis vs. Moderate/Severe NAFLD [8,13,14,17,18,24,47,48,49,50,51,52,56]. NAFLD—non-alcoholic fatty liver disease; LVEF—left ventricular ejection fraction; GLS—global longitudinal strain; GSRS—global systolic strain rate; SRearly—strain rate in early phase of diastole; SRlate—strain rate in late phase of diastole; GCS—global circumferential strain; GAS—global area strain; GRS—global radial strain.

The GRS strain parameter was evaluated in a total of two studies [13,55]. The pooled studies showed an overall MD of 6.836 (95% CI 1.065, 12.607). Substantial heterogeneity was reported with an I^2^ = 63.44% and *p*-value = 0.098 (Figure 2).

### 3.9. Strain Assessment in Diabetes Mellitus (DM) Patients vs. DM + NAFLD Patients

We evaluated LVEF across four studies, which compared DM patients to subjects having DM and NAFLD [13,49,52,55]. The analysis of studies revealed an overall MD of 0.850 (95% CI −0.989, 2.690). Moderate heterogeneity was observed, with an I^2^ = 48.05% and a *p*-value = 0.125.

GLS was examined in four studies [13,49,52,55], demonstrating an overall MD of −1.740 (95% CI −3.252, −0.228). Considerable heterogeneity was reported with an I^2^ = 84.49% and *p*-value < 0.001.

GSRS was assessed in two studies [49,52], showing an overall MD of −0.027 (95% CI −0.072, 0.018). Heterogeneity was reported as not important with an I^2^ = 0% and *p*-value = 0.842.

SRearly parameter was analyzed in two studies [49,52], revealing an overall MD of 0.093 (95% CI 0.029, 0.157). Negligible heterogeneity was observed, with an I^2^ = 0% and a *p*-value = 0.890.

SRlate parameter was assessed in two studies [49,52], demonstrating an overall MD of 0.006 (95% CI −0.170, 0.182). Substantial heterogeneity was reported with an I^2^ = 85.11% and *p*-value = 0.010.

GCS was evaluated in two studies [13,55], demonstrating an overall MD of −1.244 (95% CI −2.204, −0.284). Non-significant heterogeneity was reported with an I^2^ = 0% and *p*-value = 0.992.

GAS was evaluated in a total of two studies [13,55]. The pooled studies analyzing GAS revealed an overall MD of −3.422 (95% CI −4.645, −2.199). Heterogeneity was reported as insignificant with an I^2^ = 0% and *p*-value = 0.851.

GRS was assessed in two studies [13,55], demonstrating an overall MD of 4.551 (95% CI 0.704, 8.397). Substantial heterogeneity was reported with an I^2^ = 57.04% and *p*-value = 0.127.

### 3.10. Strain and LVEF Assessment in Patients with DM Patients vs. DM + Mild NAFLD

We evaluated LVEF across two studies that compared a DM group to patients with DM and mild NAFLD [13,55]. The analysis of studies revealed an overall MD of 0.547 (95% CI −1.456, 2.550). Unimportant heterogeneity was observed, with an I^2^ = 0% and a *p*-value = 0.330.

GLS was evaluated in two studies [13,55], demonstrating an overall MD of −0.866 (95% CI −1.963, 0.231). Unimportant heterogeneity was reported with an I^2^ = 0% and *p*-value = 0.611.

GCS was assessed in two studies [13,55], showing an overall MD of −0.133 (95% CI −1.317, 1.052). Heterogeneity was described as unimportant and was reported with an I^2^ = 0% and *p*-value = 0.943.

GAS was assessed in two studies [13,55], demonstrating an overall MD of −0.971 (95% CI −2.886, 0.943). Moderate heterogeneity was reported with an I^2^ = 34.58% and *p*-value = 0.216.

For the evaluation of the GRS strain parameter, we evaluated a total of two studies [13,55]. Analysis assessing this strain parameter demonstrated an overall MD of 0.238 (95% CI −2.936, 3.411). Heterogeneity was reported as not significant with an I^2^ = 0% and *p*-value = 0.740.

### 3.11. Strain and LVEF Assessment in DM Patients vs. DM + Moderate/Severe NAFLD

We evaluated LVEF across two studies, which compared a control group with DM to a group with DM and moderate to severe NAFLD [13,55]. The analysis of studies evaluating the LVEF in control subjects with DM vs. subjects having DM and moderate to severe NAFLD revealed an overall MD of 0.998 (95% CI −0.961, 2.957). Unimportant heterogeneity was observed, with an I^2^ = 0% and a *p*-value = 0.532.

GLS was evaluated in a total of two studies [13,55], demonstrating an overall MD of −4.385 (95% CI −5.400, −3.369). We described an unimportant heterogeneity with an I^2^ = 0% and *p*-value = 0.436.

In a total of two studies comparing subjects with DM with subjects having moderate to severe NAFLD and DM [13,55], we assessed the GCS, revealing an overall MD of −2.221 (95% CI −3.307, −1.135). Negligible heterogeneity was reported with an I^2^ = 0% and *p*-value = 0.901.

GAS was assessed in two studies [13,55], demonstrating an overall MD of −4.919 (95% CI −6.320, −3.519). No significant heterogeneity was observed with an I^2^ = 0% and *p*-value = 0.758.

GRS was evaluated in a total of two studies [13,55], showing an overall MD of 7.167 (95% CI 2.475, 11.859). We described substantial heterogeneity with an I^2^ = 71.93% and *p*-value = 0.059.

### 3.12. Strain and LVEF Assessment in Patients with DM and Mild NAFLD vs. Patients with DM and Moderate/Severe NAFLD

We evaluated the LVEF among two studies that compared a group with DM and mild NAFLD to a group with DM and moderate to severe NAFLD [13,55]. The analysis of studies evaluating the LVEF in these two groups revealed an overall MD of 0.585 (95% CI −1.231, 2.402). Unimportant heterogeneity was observed, with an I^2^ = 0% and a *p*-value = 0.629.

GLS was assessed in two studies [13,55]. The pooled studies for the analysis assessing this strain parameter demonstrated an overall MD of −3.684 (95% CI −4.956, −2.412). Unimportant heterogeneity was reported with an I^2^ = 0% and *p*-value = 0.847.

GCS was analyzed in two studies [13,55], showing an overall MD of −2.126 (95% CI −3.335, −0.917). We described an irrelevant heterogeneity with an I^2^ = 0% and *p*-value = 0.862.

For the evaluation of the GAS parameter, we examined two studies [13,55], demonstrating an overall MD of −3.939 (95% CI −6.288, −1.591). Moderate heterogeneity was reported with an I^2^ = 44.62% and *p*-value = 0.179.

**Figure 3:**
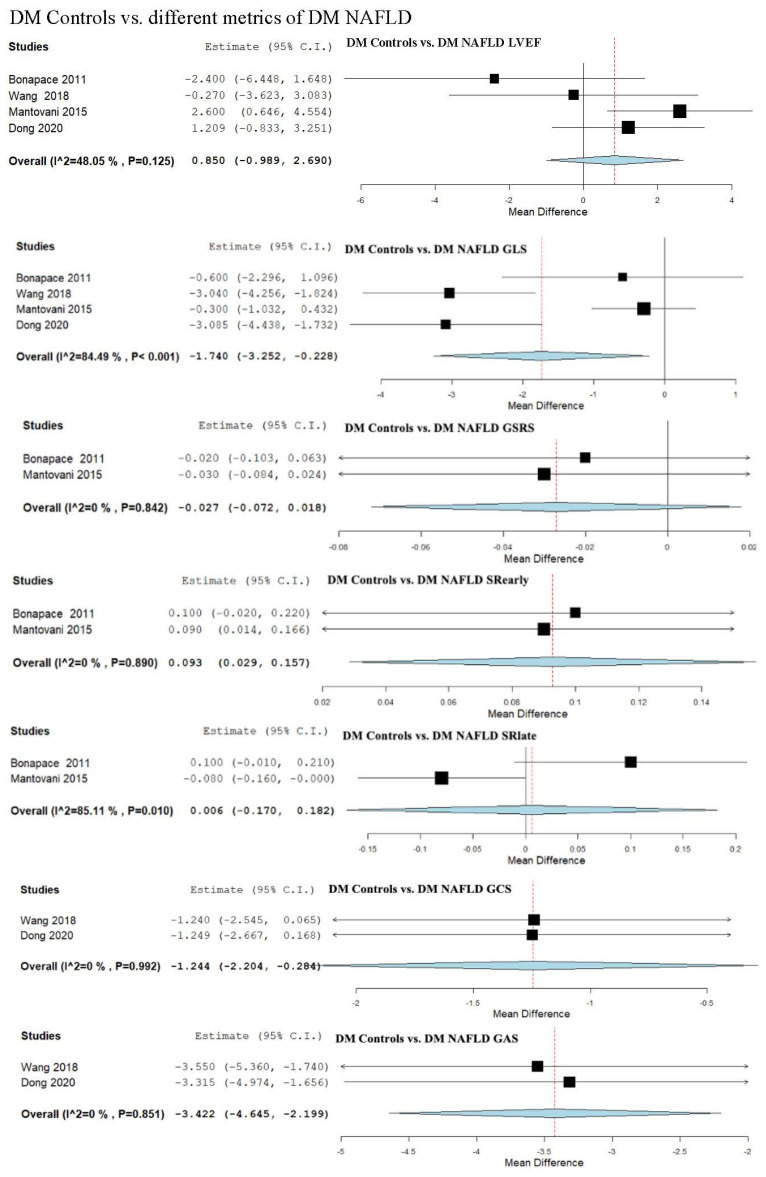

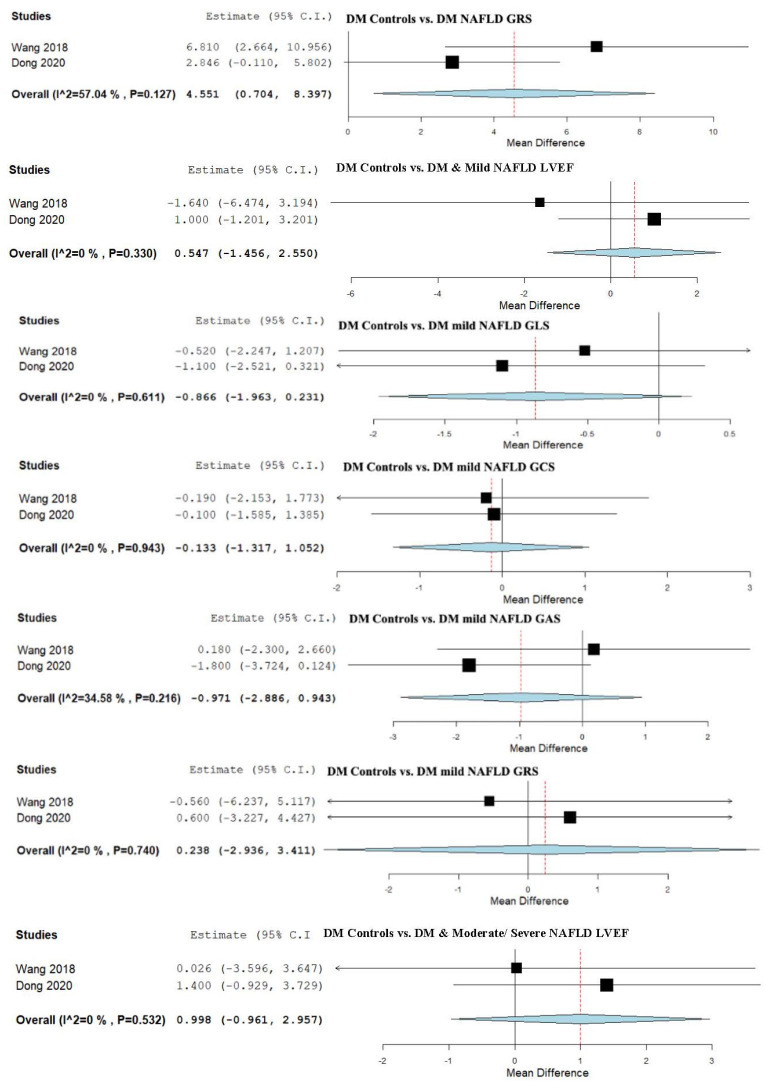

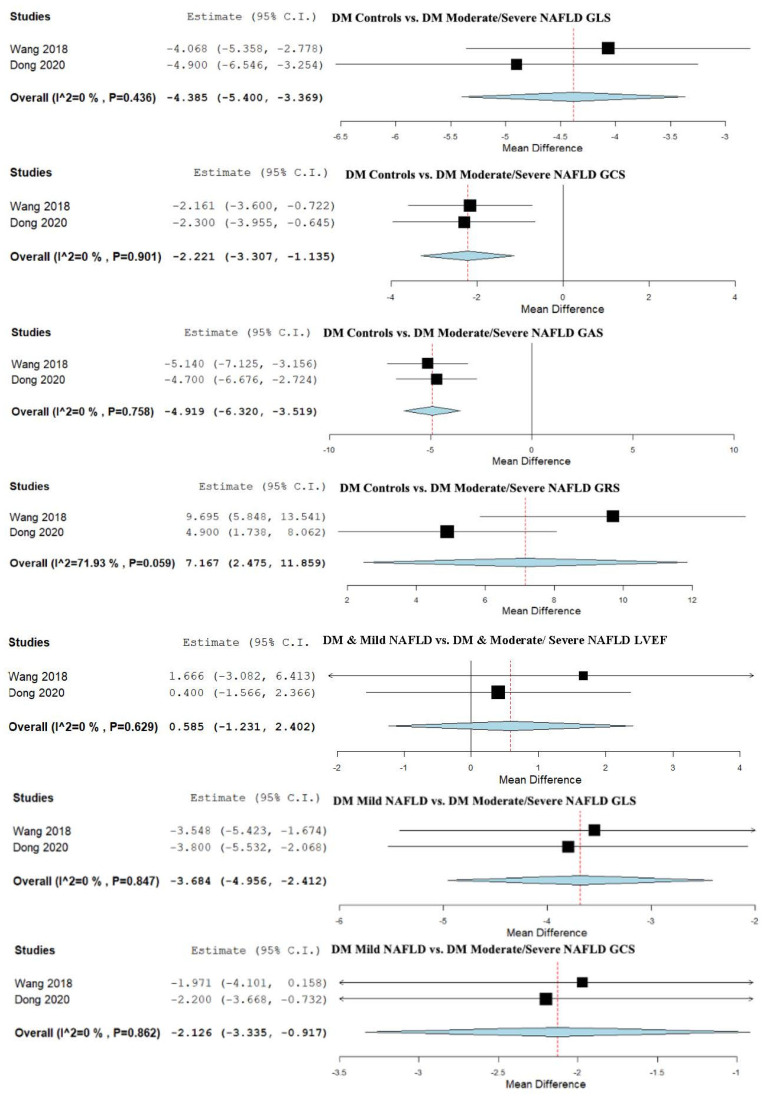

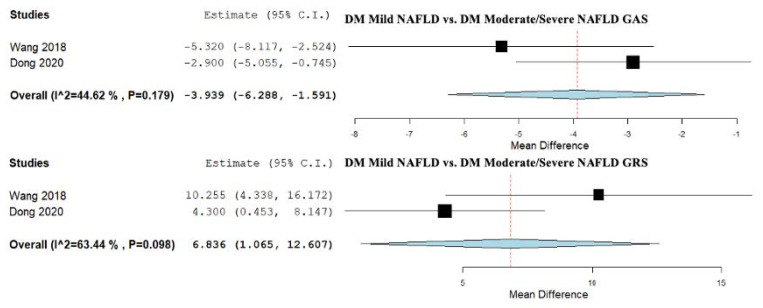
Studies evaluating myocardial strain parameters in DM-Controls vs. DM-NAFLD, DM-Controls vs. DM-Mild NAFLD, DM-Controls vs. DM-Moderate/Severe NAFLD, and DM-Mild NAFLD vs. DM-Moderate/Severe NAFLD [13,49,52,55]. NAFLD—non-alcoholic fatty liver disease; DM—diabetes mellitus; LVEF—left ventricular ejection fraction; GLS—global longitudinal strain; GSRS—global systolic strain rate; SRearly—strain rate in early phase of diastole; SRlate—strain rate in late phase of diastole; GCS—global circumferential strain; GAS—global area strain; GRS—global radial strain.

We compared two studies to evaluate the GRS [13,55]. The emerging studies for the analysis assessing this strain parameter demonstrated an overall MD of 6.836 (95% CI 1.065, 12.607). Potentially substantial heterogeneity was reported with an I^2^ = 63.44% and *p*-value = 0.098 (Figure 3).

### 3.13. Strain and LVEF Assessment in Controls vs. NASH

We evaluated LVEF across two studies that compared a control group to NASH patients [50,53]. The analysis of studies revealed an overall MD of 1.020 (95% CI −3.186, 5.226). Substantial heterogeneity was observed, with an I^2^ = 81.28% and a *p*-value = 0.021.

We evaluated two studies [50,53], to analyze the GLS parameter, demonstrating an overall MD of −3.568 (95% CI −6.257, −0.879). Substantial heterogeneity was reported with an I^2^ = 85.9% and *p*-value = 0.008.

GSRS was evaluated in a total of two studies [50,53], revealing an overall MD of −0.410 (95% CI −0.791, −0.029). Substantial heterogeneity was reported with an I^2^ = 86.64% and *p*-value = 0.006.

SRearly was analyzed in a total of two studies [50,53], showing an overall MD of 0.521 (95% CI −0.704, 1.746). Heterogeneity must be considered with an I^2^ = 98.55% and *p*-value < 0.001.

SRlate was evaluated in a total of two studies [50,53], showing an overall MD of 0.271 (95% CI −0.346, 0.888). Heterogeneity must be considered with an I^2^ = 97.59% and *p*-value < 0.001.

### 3.14. Strain and LVEF Assessment in Non-Alcoholic Fatty Liver (NAFL) vs. NASH

We evaluated LVEF across three studies, which compared NAFL patients to NASH patients [50,54,57]. The analysis of studies revealed an overall MD of 0.015 (95% CI −2.177, 2.207). Unimportant heterogeneity was observed, with an I^2^ = 0% and a *p*-value = 0.295.

**Figure 4:**
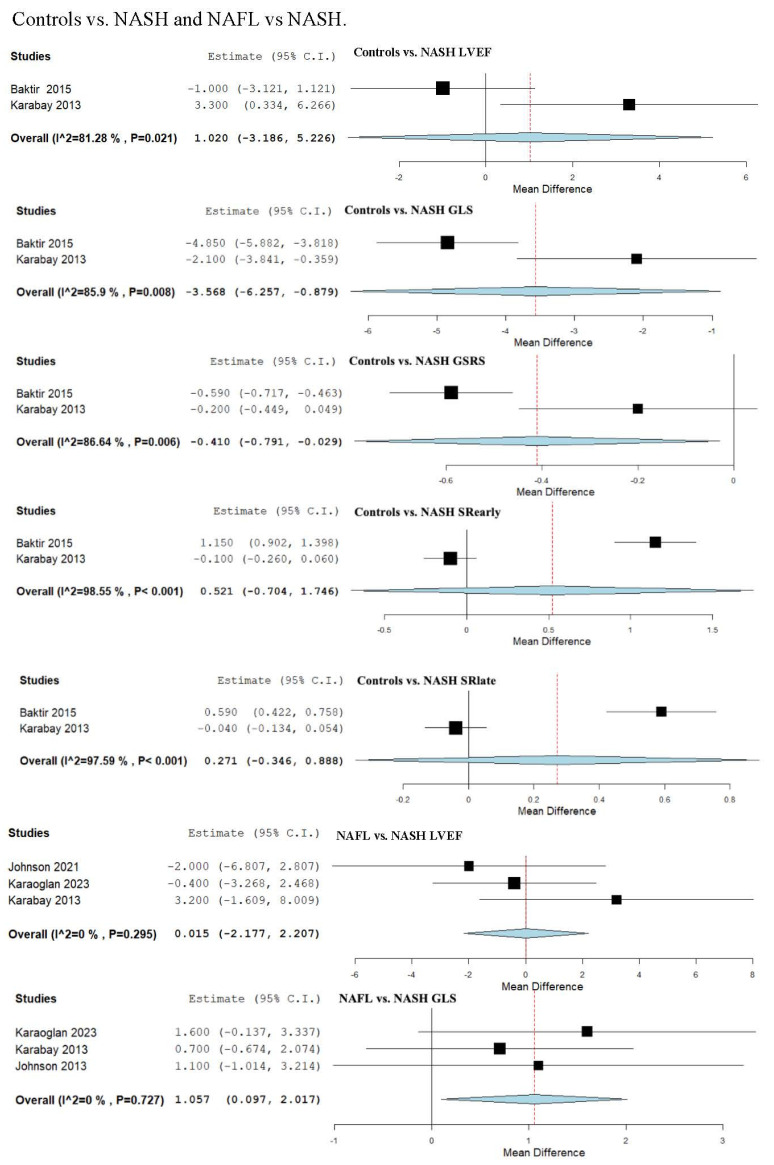
Studies evaluating myocardial strain parameters in Controls vs. NASH and NAFL vs. NASH [50,53,54,57]. NAFL—non-alcoholic fatty liver; NASH—non-alcoholic steatohepatitis. LVEF—left ventricular ejection fraction; GLS—global longitudinal strain; GSRS—global systolic strain rate; SRearly—strain rate in early phase of diastole; SRlate—strain rate in late phase of diastole.

GLS was examined in three studies [50,54,57], demonstrating an overall MD of 1.057 (95% CI 0.097, 2.017). Non-significant heterogeneity was reported with an I^2^ = 0% and *p*-value = 0.727 (Figure 4).

### 3.15. LVEF Assessment in DM NAFL vs. NAFL

**Figure 5:**
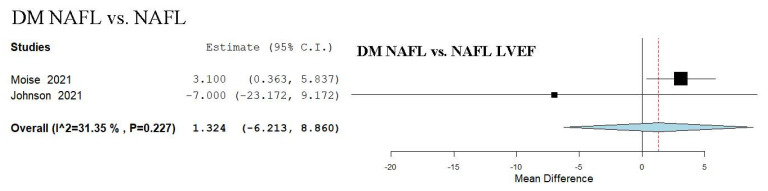
Study evaluating echocardiographic parameters including LVEF in DM NAFL vs. NAFL [47,54]. NAFL—non-alcoholic fatty liver; DM—diabetes mellitus; LVEF—left ventricular ejection fraction.

We evaluated the LVEF parameter across two studies, which compared NAFL patients and DM to a group with only NAFL [47,54]. The analysis of studies revealed an overall MD of 1.324 (95% CI −6.213, 8.860). Moderate heterogeneity was observed, with an I^2^ = 31.35% and a *p*-value = 0.227 (Figure 5).

### 3.16. Quality Assessment

The Newcastle-Ottawa Scale (NOS) quality assessment tool was utilized to appraise the methodological quality of the eligible studies included in the qualitative analysis of this systematic review and meta-analysis, as detailed in Supplementary Table S2. A total of 16 articles were assessed using the NOS criteria for cross-sectional studies. Among these, only one study received an overall rating of 5 stars [47]. Three studies were rated with 6 stars [14,48,56]. The majority, comprising ten studies, achieved an overall rating of 7 stars [13,17,18,49,50,51,52,53,54,55]. Additionally, two studies were awarded an 8-star rating [8,57].

## 4. Discussion

### 4.1. Main Findings

This systematic review and meta-analysis aimed to clarify the impact of NAFLD on subclinical myocardial function using advanced echocardiographic measures. To comprehensively assess cardiac function, this study examined all available strain parameters derived from STE, alongside LVEF as a standard indicator of systolic function [58]. Overall, our findings show that LVEF remained unchanged across NAFLD severity groups and comparisons [59]. While GLS consistently demonstrated significant reductions, indicating that GLS serves as a more sensitive marker for early systolic dysfunction than conventional LVEF, which could fail to detect subtle myocardial impairment [60]. In addition, other strain parameters, including GCS and GAS, also revealed meaningful impairment. Especially in patients with more advanced NAFLD stages. Altogether, these findings indicate a clear subclinical impact on cardiac function, with evidence of progressively worsening myocardial impairment as NAFLD severity increases and additional conditions, such as diabetes mellitus, coexist [61].

### 4.2. Controls vs. NAFLD and Severity Spectrum

Across all included studies, LVEF showed no significant difference when comparing overall NAFLD patients to healthy controls and likewise between subgroups ranging from simple steatosis to moderate/severe NAFLD. This points out that conventional systolic function remains preserved despite underlying myocardial changes. In contrast, GLS was consistently reduced in NAFLD patients compared to controls, with the reduction becoming more pronounced as disease severity increased. Patients with simple steatosis already demonstrated significantly impaired GLS, and this impairment deepened in those with moderate to severe NAFLD [62]. This gradient suggests a clear link between hepatic fat accumulation and the progressive development of subclinical, or even clinical, systolic dysfunction [63]. When considering further STE parameters: GCS, GAS, and GRS, a similar pattern emerged. For simple steatosis, these parameters mostly remained unchanged compared to controls, indicating early subclinical changes may initially be confined to longitudinal fibers. However, in moderate to severe NAFLD, significant reductions in GCS and GAS were evident, pointing towards circumferential and transmural myocardial involvement as hepatic disease advances [64,65]. Interestingly, GRS did not show significant differences in mild NAFLD but tended to increase with more advanced stages, suggesting a more uniformly radial thickening later in disease progression. Together, these findings confirm that subclinical myocardial dysfunction in NAFLD evolves progressively, with GLS being the earliest marker, followed by layered strain deterioration reflected in GCS, GAS.

This apparent discrepancy, a preserved LVEF alongside a significantly impaired GLS, is in fact the central finding and the classic signature of subclinical myocardial injury [66]. The pathophysiological reason for this is the layered nature of myocardial dysfunction. The subendocardial longitudinal fibers, which are the primary determinants of GLS, are the most vulnerable to the metabolic insults, characteristic of NAFLD, such as lipotoxicity and systemic inflammation [67]. Their impairment represents the earliest stage of cardiac damage. In contrast, LVEF is a global measure heavily influenced by circumferential and radial fibers, which are typically compromised only later in the disease process. Therefore, our findings do not show a contradiction but rather capture the precise echocardiographic profile of early, subclinical systolic dysfunction [68].

### 4.3. Diabetes Subgroups and NAFLD Severity

In patients with DM, the pattern was broadly consistent yet showed an amplified burden of myocardial strain alteration [69]. LVEF again did not differ significantly between diabetic patients with and without NAFLD, nor between those with mild versus moderate/severe NAFLD, indicating preserved global pump function. However, GLS was significantly reduced in diabetic patients with NAFLD compared to diabetic controls. Notably, when stratified by severity, moderate/severe NAFLD with diabetes demonstrated significant GLS impairment compared to mild NAFLD with diabetes. Reinforcing the progressive nature of myocardial involvement [46]. It is plausible that both DM and NAFLD exert a detrimental effect on myocardial function [70]. According to a meta-analysis published in 2021, patients with diabetes alone exhibit a significant reduction in GLS as well as in GAS, GAS and GCS when compared to a healthy control group, indicating early subclinical systolic dysfunction [71]. This may explain why, when comparing diabetic controls with patients who have diabetes and mild NAFLD, no significant additional impairment in GLS was observed, likely because baseline cardiac function is already compromised by diabetes alone [72]. However, as NAFLD severity advances within the diabetic population, we observed a significant reduction in GLS compared to diabetic controls, suggesting that the combined metabolic burden of progressive NAFLD and diabetes may compound myocardial dysfunction beyond the effect of diabetes alone [69]. Notably, in non-diabetic comparisons, early GLS changes were already evident even with mild NAFLD. For further STE parameters, GCS and GAS, no significant changes were observed in mild NAFLD within patients with DM. While moderate/severe NAFLD with DM showed consistent reductions across these parameters. Meaning that radial and circumferential fiber dysfunction, suggesting a more mid-wall fiber damage, are preceded by longitudinal impairment [73]. It seems plausible that DM acts as an important accelerator of myocardial strain impairment in NAFLD patients, emphasizing the need for vigilant cardiac assessment in this high-risk group.

### 4.4. NASH vs. NAFL and Controls

When comparing NASH to both NAFL and healthy controls, our findings revealed an unexpected but notable pattern. While LVEF remained unchanged across these groups. GLS was significantly reduced in patients with NASH compared to the control group. However, GLS was higher when compared with the NAFL group. Despite the expectation that the progression from simple steatosis to NASH, which reflects more severe hepatic injury and inflammation, would likewise be associated with greater cardiac impairment [74,75]. The observation that GLS was more significantly reduced in NAFL patients compared to those with NASH warrants further investigation to clarify this unexpected pattern. It remains necessary to determine whether this finding reflects true pathophysiological differences or is partly due to heterogeneity, small subgroup sample sizes, or methodological variation across studies [76]. Future research should therefore aim to elucidate whether potential compensatory cardiac mechanisms in response to the inflammatory state of NASH may transiently preserve myocardial function and to confirm whether such compensatory adaptations meaningfully influence strain measurements in this context. GCS, GRS, and GAS trends generally supported the interpretation that more advanced hepatic pathology is associated with greater myocardial strain impairment.

Taken together, our findings suggest that although the inflammatory progression towards NASH would typically be expected to exacerbate myocardial strain abnormalities, compensatory or confounding factors may temporarily obscure further GLS deterioration. Nonetheless, the overall pattern emerging from our systolic strain analysis, including GLS, GCS, and GAS, reveals a consistent, stepwise decline that begins with simple steatosis and becomes more pronounced with increasing hepatic severity and the presence of type 2 diabetes [77]. This trajectory underscores the complex interaction between hepatic inflammation and cardiac adaptation. However, systolic strain represents only one aspect of the cardiac cycle. Because diastolic dysfunction is a defining feature of HFpEF, a condition closely associated with NAFLD, it is equally important to investigate diastolic strain rate parameters to gain a more complete understanding of how NAFLD influences cardiac mechanics.

### 4.5. Diastolic Strain Parameter

SRearly was significantly altered only in some comparisons, notably showing changes in NAFLD patients versus controls and in the diabetic NAFLD subgroup. Suggesting subtle early diastolic dysfunction in these groups. In contrast, SRlate did not show significant modification across the analyzed groups, indicating that late diastolic myocardial mechanics remain relatively preserved in this population. Combined with our findings on systolic subclinical dysfunction and the results of the systematic review meta-analysis by Goliopoulou et al., which demonstrate diastolic impairment in NFLD patients, it becomes evident that both systolic and diastolic dysfunction may occur in this context [78]. However, whether one precedes the other or they tend to develop simultaneously remains to be clarified by future research [79].

### 4.6. Pathophysiological Mechanisms

While our analysis confirms the presence of subclinical cardiac dysfunction, the precise pathophysiological links between NAFLD and the myocardium are complex and multifactorial. The shared driver of insulin resistance is central, promoting both hepatic steatosis and deleterious ectopic fat deposition, including myocardial steatosis (cardiac lipotoxicity) and increased epicardial adipose tissue [80]. This lipotoxicity, combined with a systemic pro-inflammatory state driven by the diseased liver (particularly in NASH), leads to the release of cytokines, adipokines, and pro-fibrotic mediators [81]. This cascade results in increased oxidative stress, endothelial dysfunction, autonomic dysregulation, and ultimately, direct myocardial injury, and interstitial fibrosis, which impair myocyte contractility long before the LVEF declines [82].

### 4.7. Clinical Implications

This systematic review and meta-analysis demonstrate that NAFLD, spanning from simple steatosis to NASH, is associated with progressive subclinical LV systolic dysfunction not captured by standard LVEF assessment. The stepwise deterioration of GLS, together with consistent worsening of GCS, GAS, and GRS in more severe phenotypes, highlights the layered and progressive involvement of myocardial fibers as hepatic disease progresses [13,55]. These results highlight the importance of shifting from traditional LVEF-based assessments to more sensitive myocardial strain parameters when evaluating cardiovascular risk in patients with NAFLD [83]. This is also supported by a recently published systematic review and meta-analysis about patients with MASLD [84]. Given the progressive and often silent nature of myocardial impairment in this population, implementing STE-based evaluation, particularly GLS, GAS, GRS, and GCS in routine echocardiographic screening, could allow clinicians to detect cardiac dysfunction at a reversible stage. Such early identification would provide a critical opportunity for timely intervention, lifestyle modification, and more targeted risk stratification.

Considering these insights, our findings advocate for the broader integration of STE-derived strain analysis into everyday cardiological and hepatological practice. This approach may not only help identify high-risk patients earlier but could also support more proactive management strategies aimed at preventing progression to symptomatic heart failure. Ultimately, this paradigm shift towards advanced imaging in NAFLD patients has the potential to improve long-term cardiovascular outcomes in a population that is rapidly expanding globally.

### 4.8. Study Limitations and Strengths

Several limitations should be considered. The overall sample size, particularly for evaluating myocardial strain in NASH patients, was relatively small, limiting the study’s capacity to draw statistically significant conclusions for this parameter [50,53,54,57]. Furthermore, the heterogeneity in diagnostic methods for NAFLD across the studies, including the use of ultrasonography, liver biopsy, and CT scans, may introduce variability in the accuracy of NAFLD and NASH classifications [85]. Future studies with standardized methodologies and larger sample sizes are needed to enhance the validity and specificity of these findings, especially concerning myocardial strain differences across NAFLD and NASH groups. Variations in methodologies and heterogeneity could contribute to differing outcomes, necessitating caution when interpreting pooled data and acknowledging that some variability in the findings may stem from these differences. Most of the included studies employed a monocentric study design, which may limit the generalizability of the findings to broader and more diverse patient populations. Another limitation of this study is the disproportionate sample sizes between NAFLD patients and control groups, with smaller sample sizes in the NAFLD group potentially reducing the statistical power and affecting the reliability of comparisons between the groups [86]. Furthermore, it is important to acknowledge that STE-derived strain measurements are inherently dependent on several technical and patient-related factors. These include the experience and proficiency of the operator, the overall quality of the echocardiographic images obtained, and individual patient characteristics such as body habitus or thoracic anatomy [87]. These variables may introduce measurement variability and limit comparability across studies, underscoring the need for standardized imaging protocols and training to ensure reproducibility and diagnostic accuracy in clinical and research settings. This meta-analysis is based on studies that almost exclusively used the now-outdated NAFLD terminology. Future research will be essential to confirm and extend our findings within cohorts defined by the new, more precise metabolic-dysfunction-associated steatotic liver disease (MASLD) criteria [88].

However, this study has notable strengths, including a comprehensive sample that includes participants from diverse racial and ethnic backgrounds, enhancing the generalizability of results across varied populations. This review addresses a clinically significant topic, especially given the rapid worldwide rise in NAFLD prevalence, coupled with its link to elevated morbidity and mortality due to cardiovascular diseases [89]. We applied an extensive search strategy utilizing multiple medical databases, ensuring a systematic and comprehensive evaluation of the studies. Additionally, by systematically excluding articles that did not meet specific methodological criteria, such as studies without appropriate control groups or lacking echocardiographic assessment, the study ensures high-quality evidence.

## 5. Conclusions and Future Directions

This meta-analysis demonstrates that NAFLD is associated with subclinical myocardial dysfunction, particularly in advanced disease stages and in patients with diabetes. While LVEF remained preserved, GLS showed consistent and significant reductions across all NAFLD severities, highlighting its role as a sensitive marker of early systolic impairment [90]. GCS, GRS, and GAS were also impaired in more severe NAFLD, indicating progressive myocardial involvement. These findings support the integration of STE-derived strain parameters into routine cardiac assessments for NAFLD patients to enable early detection and timely intervention. Future studies should focus on standardizing diagnostic approaches, exploring whether early identification of cardiac changes can improve long-term outcomes, and determining which therapeutic strategies may effectively reverse or halt the progression of subclinical myocardial dysfunction in patients with NAFLD.

## Figures and Tables

**Table 1:** Summary of included studies evaluating subclinical systolic dysfunction in NAFLD.

Study (Author, Year)	Country	Study Design	Total Subjects (N)	NAFLD Diagnosis Method	Key Comparison Groups	Key GLS Finding	NOS Score (/10)
Bonapace S et al., 2011 [49]	Italy	Cross-sectional	50	Ultrasound (US)	Controls vs. NAFLD (all DM)	Reduced in NAFLD	7
Karabay C et al., 2013 [50]	Turkey	Cross-sectional	76	Biopsy	Controls vs. Simple Steatosis vs. NASH	Reduced in all NAFLD groups	7
Khoshtbaten M et al., 2015 [18]	Iran	Cross-sectional	60	US	Controls vs. NAFLD	Reduced in NAFLD	7
Baktir A et al., 2015 [53]	Turkey	Cross-sectional	56	Biopsy	Controls vs. NASH	Reduced in NASH	7
Mantovani A et al., 2015 [52]	Italy	Cross-sectional	222	US	DM Controls vs. DM NAFLD	Reduced in NAFLD	7
VanWagner L et al., 2015 [51]	USA	Cross-sectional	2713	CT	Controls vs. NAFLD	Reduced in NAFLD	7
Wang Q et al., 2018 [13]	China	Cross-sectional	120	US	Controls vs. DM vs. DM + NAFLD	Stepwise worsening with NAFLD severity	7
Zamirian M et al., 2018 [8]	Iran	Cross-sectional	60	Biopsy	Controls vs. NAFLD	Reduced in NAFLD	8
Dong Y. et al., 2020 [55]	China	Cross-Fsectional	97	US	DM Controls vs. DM Mild vs. DM Mod/Sev NAFLD	Progressive impairment with NAFLD severity	7
Chiu L et al., 2020 [14]	USA	Cross-sectional	2356	CT	Controls vs. NAFLD	Reduced in NAFLD; exacerbated by DM	6
Johnson P et al., 2021 [54]	USA	Cross-sectional	33	Biopsy	NAFL vs. NASH (with/without DM)	Reduced in NAFLD patients	7
Moise C et al., 2021 [47]	Romania	Cross-sectional	159	US	Controls vs. NAFLD vs. NAFLD + DM	Reduced in NAFLD	5
Sewan H et al., 2021 [17]	Iraq	Cross-sectional	60	US	Controls vs. NAFLD	Reduced in NAFLD	7
Ismaiel A et al., 2022 [56]	Romania	Cross-sectional	75	US	Controls vs. MAFLD	Worsened with NAFLD severity/fibrosis	6
Lai Y et al., 2022 [48]	Taiwan	Cross-sectional	2161	US	Controls vs. NAFLD (low/high fibrosis)	Reduced in NAFLD	6
Karaoğlan B et al., 2023 [57]	Turkey	Cross-sectional	61	Biopsy	NAFL vs. NASH	Reduced in NAFLD patients	8

Abbreviations: NAFLD = non-alcoholic fatty liver disease; NASH = non-alcoholic steatohepatitis; US = ultrasound; CT = computed tomography; DM = diabetes mellitus; DM + NAFLD = diabetes mellitus with coexisting NAFLD; Mod/Sev NAFLD = moderate/severe NAFLD; GLS = global longitudinal strain; NOS = Newcastle–Ottawa Scale; N = number of study participants.

**Table 2:** Main meta-analysis findings.

Comparison Group	LVEF (%) MD (95% CI)	GLS (%) MD (95% CI)	GCS (%) MD (95% CI)	GAS (%) MD (95% CI)	GRS (%) MD (95% CI)
Controls vs. NAFLD (Overall)	0.359 (−0.078, 0.795)	−2.043 (−2.868, −1.218)	−1.415 (−2.893, 0.064)	−3.706 (−4.999, −2.413)	7.407 (−1.852, 16.666)
Controls vs. Simple Steatosis	0.300 (−1.529, 2.129)	−2.253 (−3.502, −1.004)	−0.851 (−2.575, 0.872)	−1.383 (−2.944, 0.178)	2.128 (−1.927, 6.184)
Controls vs. Moderate/Severe NAFLD	0.619 (−1.359, 2.596)	−5.828 (−7.496, −4.160)	−3.111 (−4.630, −1.593)	−5.231 (−6.707, −3.756)	9.936 (−0.141, 20.013)
Simple Steatosis vs. Moderate/Severe NAFLD	0.585 (−1.231, 2.402)	−3.684 (−4.956, −2.412)	−2.126 (−3.335, −0.917)	−3.939 (−6.288, −1.591)	6.836 (1.065, 12.607)
DM Controls vs. DM NAFLD	0.850 (−0.989, 2.690)	−1.740 (−3.252, −0.228)	−1.244 (−2.204, −0.284)	−3.422 (−4.645, −2.199)	4.551 (0.704, 8.397)
Controls vs. NASH	1.020 (−3.186, 5.226)	−3.568 (−6.257, −0.879)	NA	NA	NA
NAFL vs. NASH	0.015 (−2.177, 2.207)	1.057 (0.097, 2.017)	NA	NA	NA

All values are Mean Difference (MD) with 95% Confidence Intervals (CI). NA: Not Assessed (insufficient data from included studies for this comparison). LVEF: Left Ventricular Ejection Fraction; GLS: Global Longitudinal Strain; GCS: Global Circumferential Strain; GAS: Global Area Strain; GRS: Global Radial Strain.

## Data Availability

The analyzed data were extracted from the cited original articles as outlined in Supplementary Table S1.

## Supplementary Materials

The following supporting information can be downloaded at: https://www.mdpi.com/article/10.3390/biomedicines13122908/s1, Search Strategy—Supplementary Material S1; Studies evaluating STE in NAFLD & NOS Quality Assessment of Cross-sectional Studies—Supplementary Tables S1 and S2; Additional STE parameters—Supplementary Figures S1–S13.
