# Supplementary material for: When the Liver Echoes to the Heart: Assessing Subclinical Cardiac Dysfunction in NAFLD Using Speckle Tracking Echocardiography—A Systematic Review and Meta-Analysis
Source: Biomedicines. 2025 Nov 27;13(12):2908. doi: 10.3390/biomedicines13122908 (PMC12730947; doi:10.3390/biomedicines13122908)
Supplement: Supplementary file 1 [file biomedicines-13-02908-s001.zip › Supplementary Figures.pdf]

Supplementary Figures

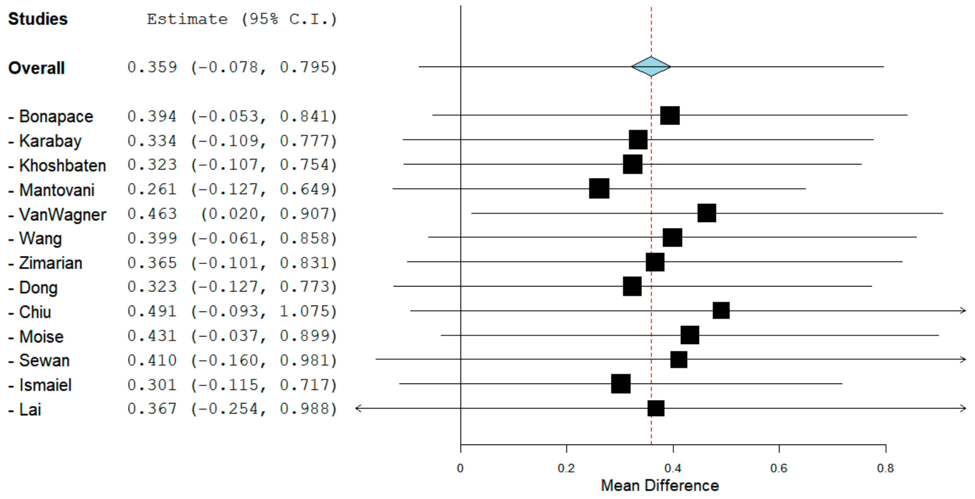

Supplementary Figure S1. Leave-one-out sensitivity analysis for LVEF in NAFLD vs. Controls

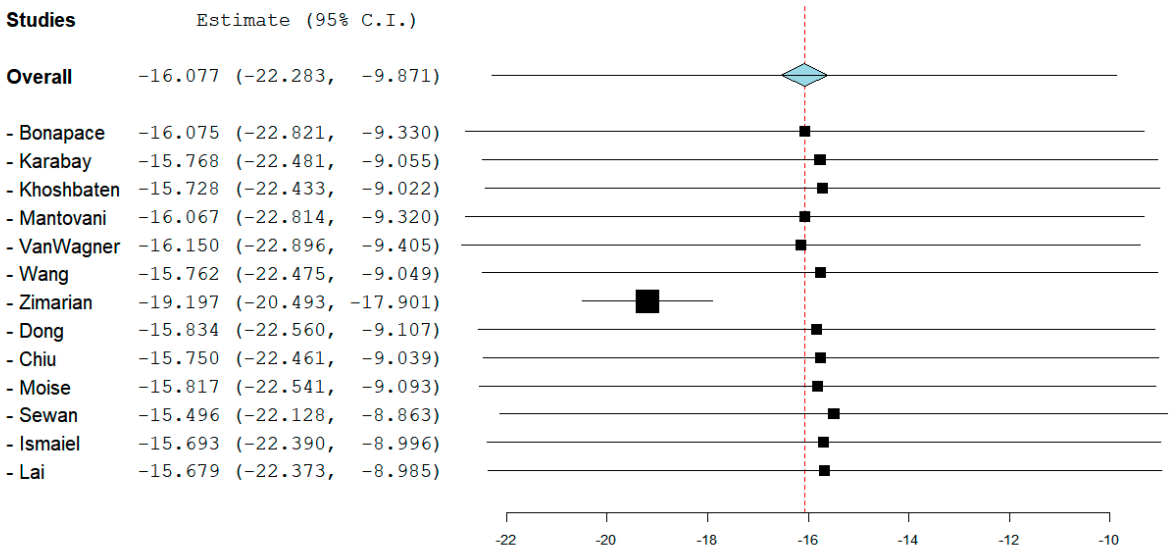

Supplementary Figure S2. Leave-one-out sensitivity analysis for GLS in NAFLD vs. Controls

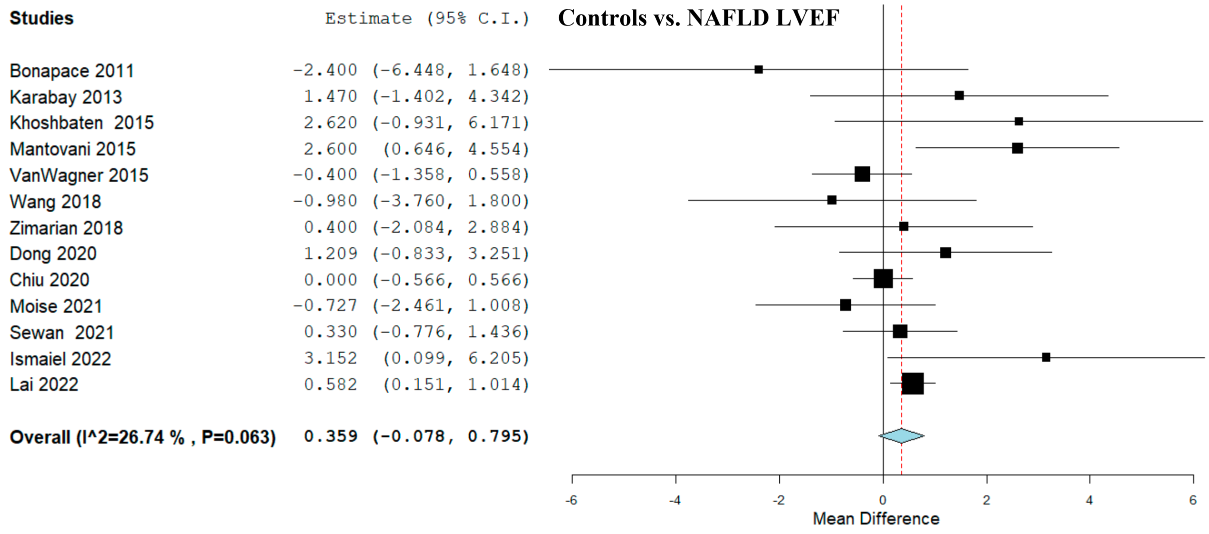
Studies
Estimate (95% C.I.)
Controls vs. NAFLD LVEF
Bonapace 2011 -2.400 (-6.448, 1.648)
Karabay 2013 1.470 (-1.402, 4.342)
Khoshbaten 2015 2.620 (-0.931, 6.171)
Mantovani 2015 2.600 (0.646, 4.554)
VanWagner 2015 -0.400 (-1.358, 0.558)
Wang 2018 -0.980 (-3.760, 1.800)
Zimarian 2018 0.400 (-2.084, 2.884)
Dong 2020 1.209 (-0.833, 3.251)
Chiu 2020 0.000 (-0.566, 0.566)
Moise 2021 -0.727 (-2.461, 1.008)
Sewan 2021 0.330 (-0.776, 1.436)
Ismaiel 2022 3.152 (0.099, 6.205)
Lai 2022 0.582 (0.151, 1.014)
Overall (I^2=26.74 % , P=0.063) 0.359 (-0.078, 0.795)
-6
-4
-2
0
2
4
6
Mean Difference

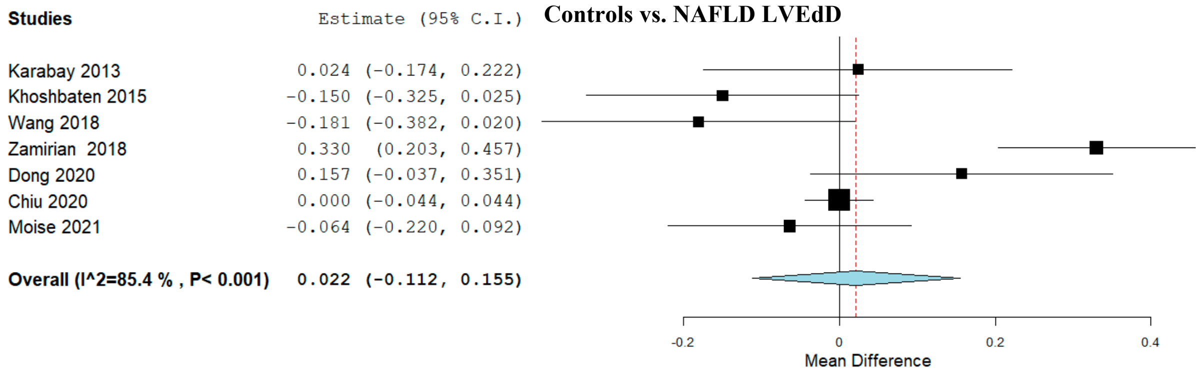
Studies
Estimate (95% C.I.)
Controls vs. NAFLD LVEdD
Karabay 2013 0.024 (-0.174, 0.222)
Khoshbaten 2015 -0.150 (-0.325, 0.025)
Wang 2018 -0.181 (-0.382, 0.020)
Zamirian 2018 0.330 (0.203, 0.457)
Dong 2020 0.157 (-0.037, 0.351)
Chiu 2020 0.000 (-0.044, 0.044)
Moise 2021 -0.064 (-0.220, 0.092)
Overall (I^2=85.4 % , P< 0.001) 0.022 (-0.112, 0.155)
-0.2
0
0.2
0.4
Mean Difference

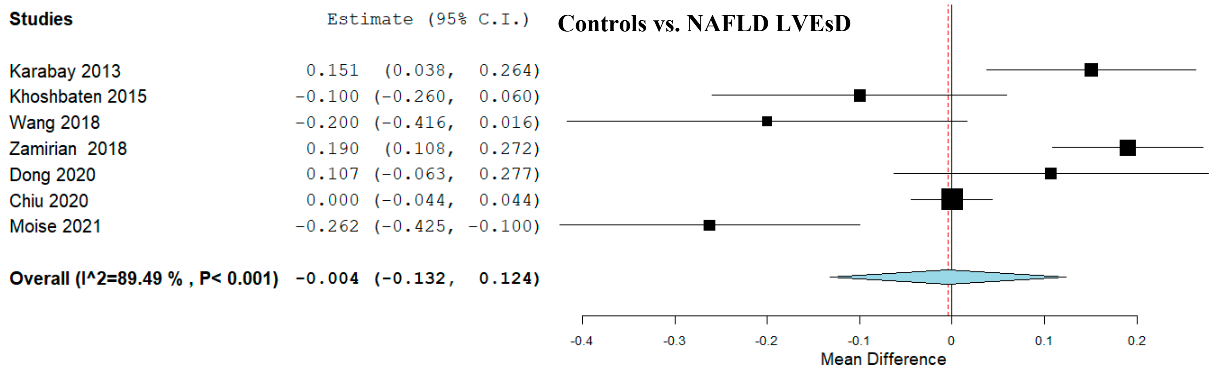
Studies
Estimate (95% C.I.)
Controls vs. NAFLD LVEsD
Karabay 2013 0.151 (0.038, 0.264)
Khoshbaten 2015 -0.100 (-0.260, 0.060)
Wang 2018 -0.200 (-0.416, 0.016)
Zamirian 2018 0.190 (0.108, 0.272)
Dong 2020 0.107 (-0.063, 0.277)
Chiu 2020 0.000 (-0.044, 0.044)
Moise 2021 -0.262 (-0.425, -0.100)
Overall (I^2=89.49 % , P< 0.001) -0.004 (-0.132, 0.124)
-0.4
-0.3
-0.2
-0.1
0
0.1
0.2
Mean Difference

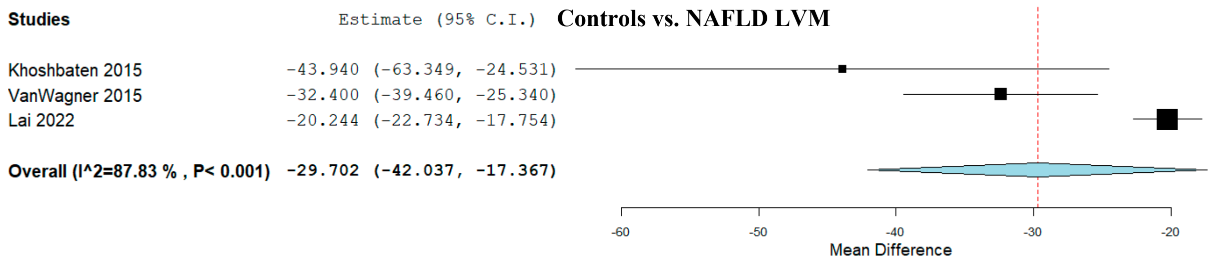
Studies
Estimate (95% C.I.)
Controls vs. NAFLD LVM
Khoshbaten 2015 -43.940 (-63.349, -24.531)
VanWagner 2015 -32.400 (-39.460, -25.340)
Lai 2022 -20.244 (-22.734, -17.754)
Overall (I^2=87.83 % , P< 0.001) -29.702 (-42.037, -17.367)
-60
-50
-40
-30
-20
Mean Difference

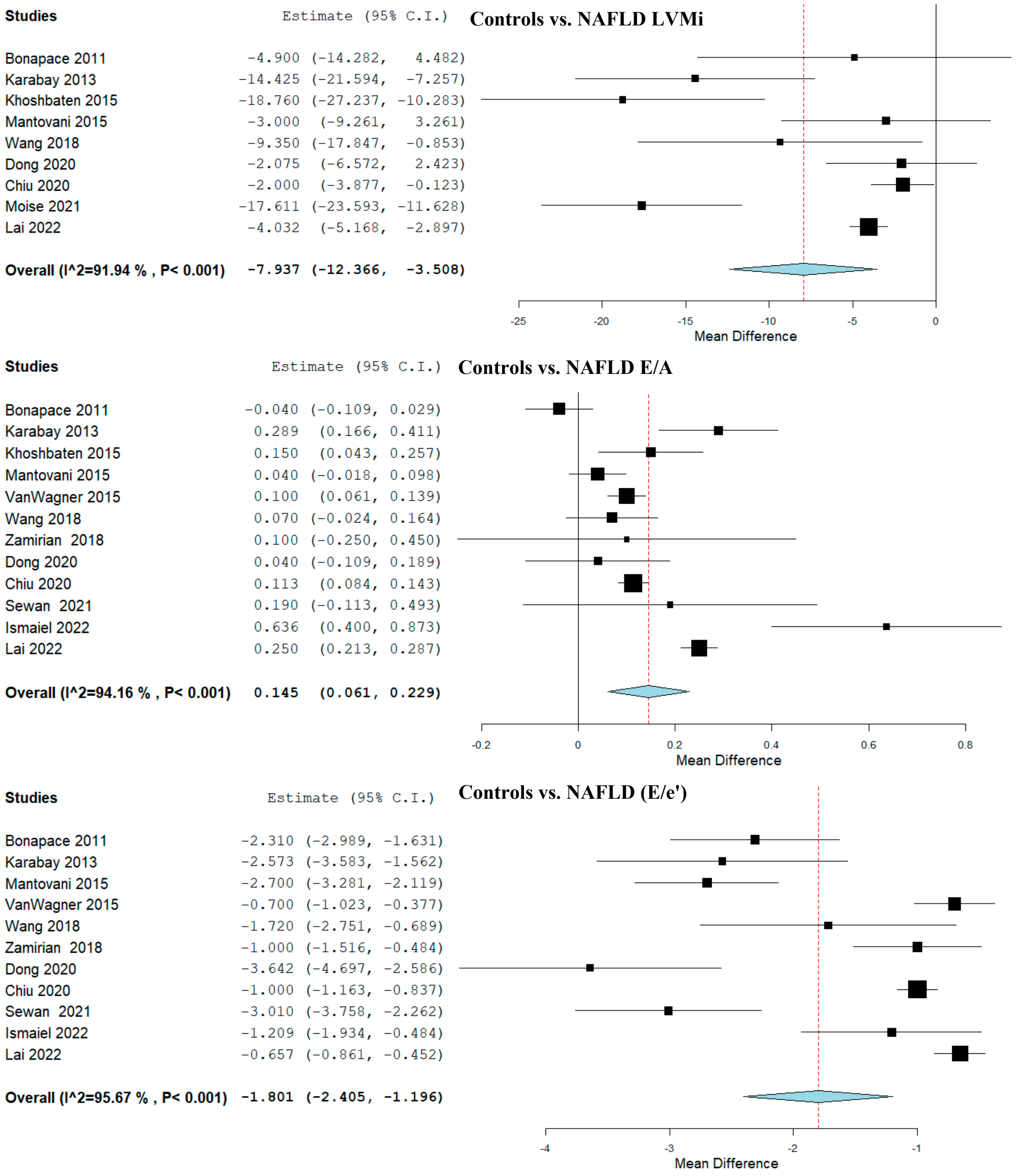

Supplementary Figure S3. Studies evaluating additional echocardiographic parameters, including LVEF, E/A, E/e', LVEdD, LVEsD, LVM, and LVMi in Controls vs. NAFLD.
A—late diastolic filling velocity; E—early diastolic filling velocity; e´—early diastolic mitral annular velocity; LVEF—Left ventricular ejection fraction; LVEdD—Left ventricular end-diastolic diameter; LVEsD—Left ventricular end-systolic diameter; LVM—Left ventricular mass; LVMi—Left ventricular mass index; NAFL—Non-alcoholic fatty liver; NAFLD—Non-alcoholic liver disease.

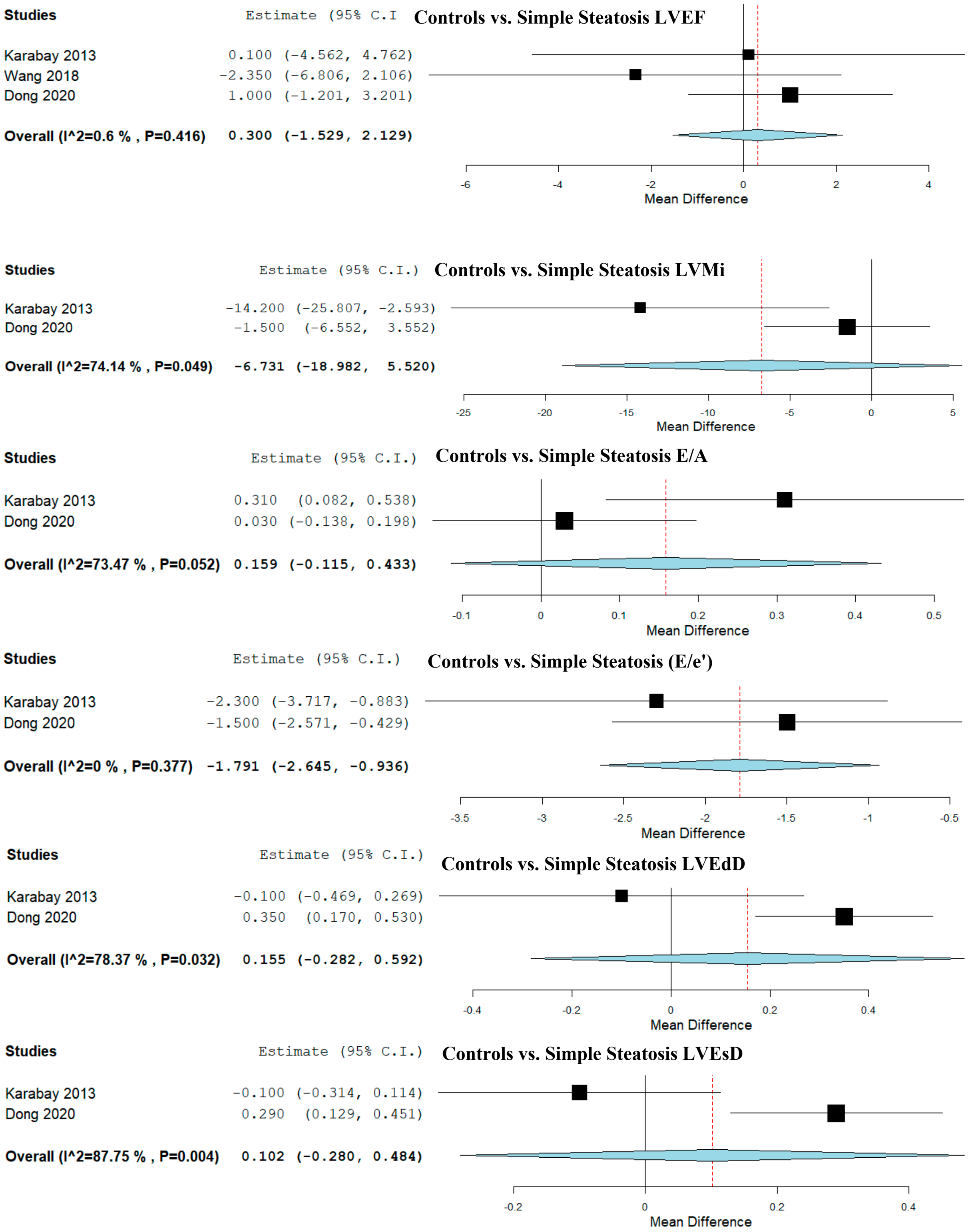

Supplementary Figure S4. Studies evaluating additional echocardiographic parameters including: LVEF, E/A, E/e', LVEdD, LVEsD, LVMi in Controls vs. Simple Steatosis.

A- late diastolic filling velocity; E- early diastolic filling velocity; e´- early diastolic mitral annular velocity; LVEF- Left ventricular ejection fraction; LVEdD- Left ventricular end-diastolic diameter; LVEsD- Left ventricular end-systolic diameter; LVMi- Left ventricular mass index.

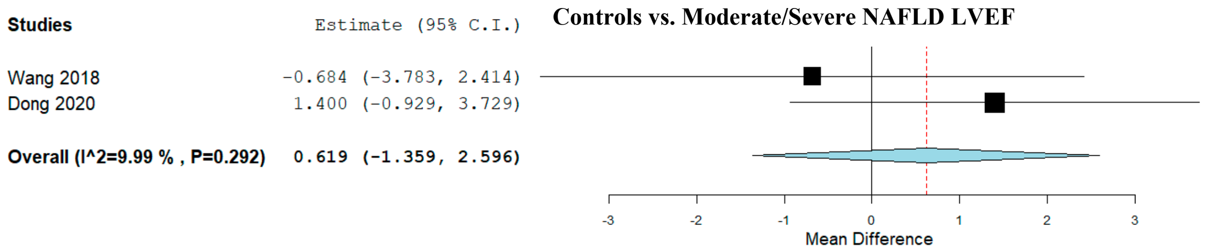

Supplementary Figure S5. Studies evaluating additional echocardiographic parameter including: LVEF in Controls vs. Moderate/Severe NAFLD.
LVEF- Left ventricular ejection fraction; NAFLD- Non-alcoholic liver disease.

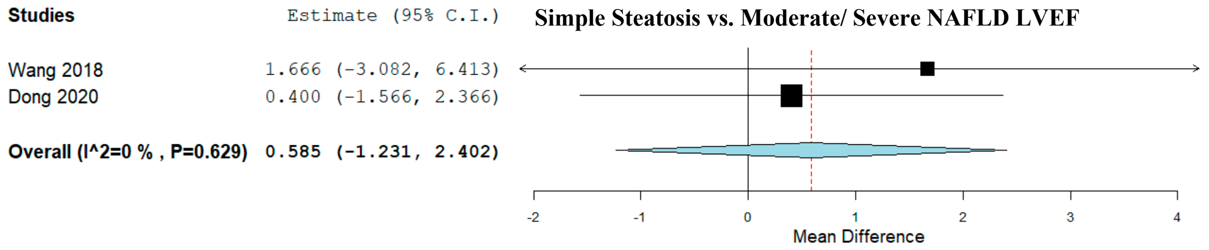

Supplementary Figure S6. Studies evaluating additional echocardiographic parameter including: LVEF in Simple Steatosis vs. Moderate/Severe NAFLD.
LVEF- Left ventricular ejection fraction; NAFLD- Non-alcoholic liver disease.

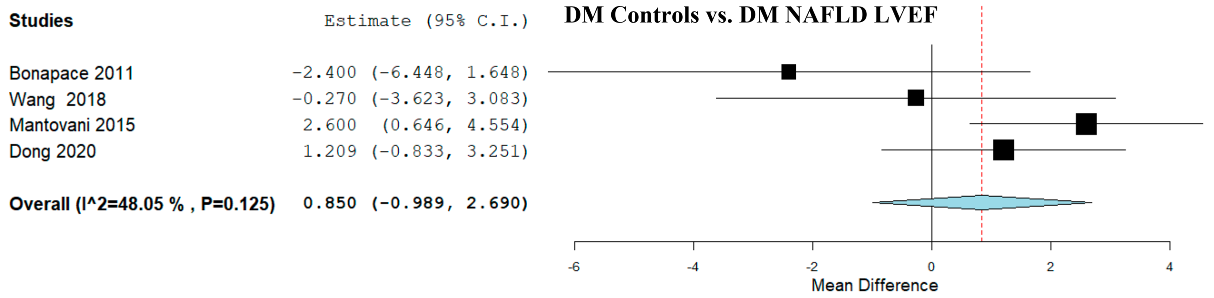

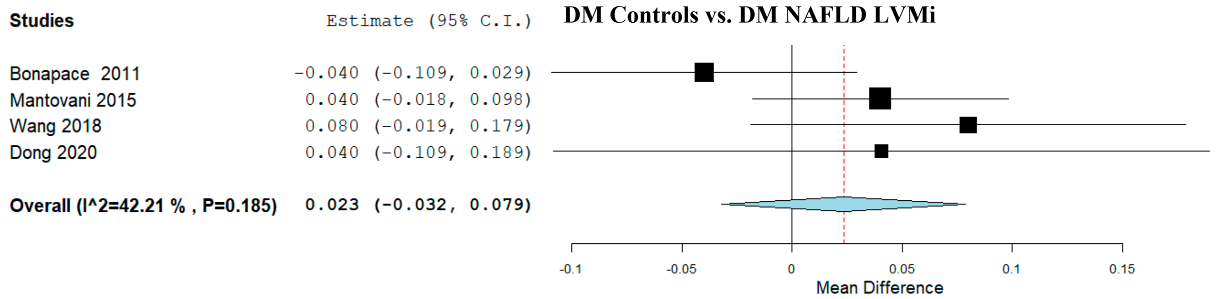

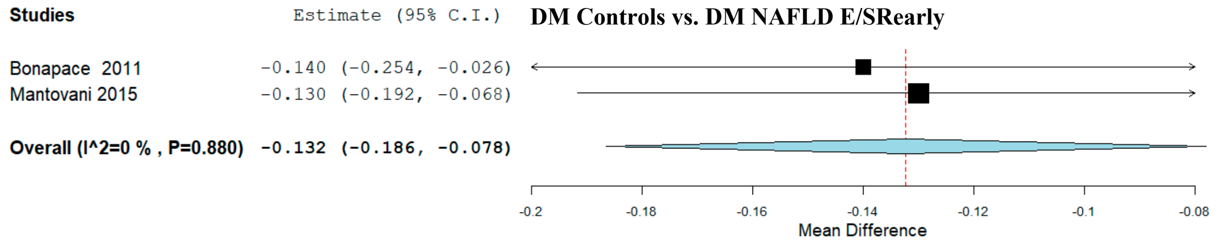

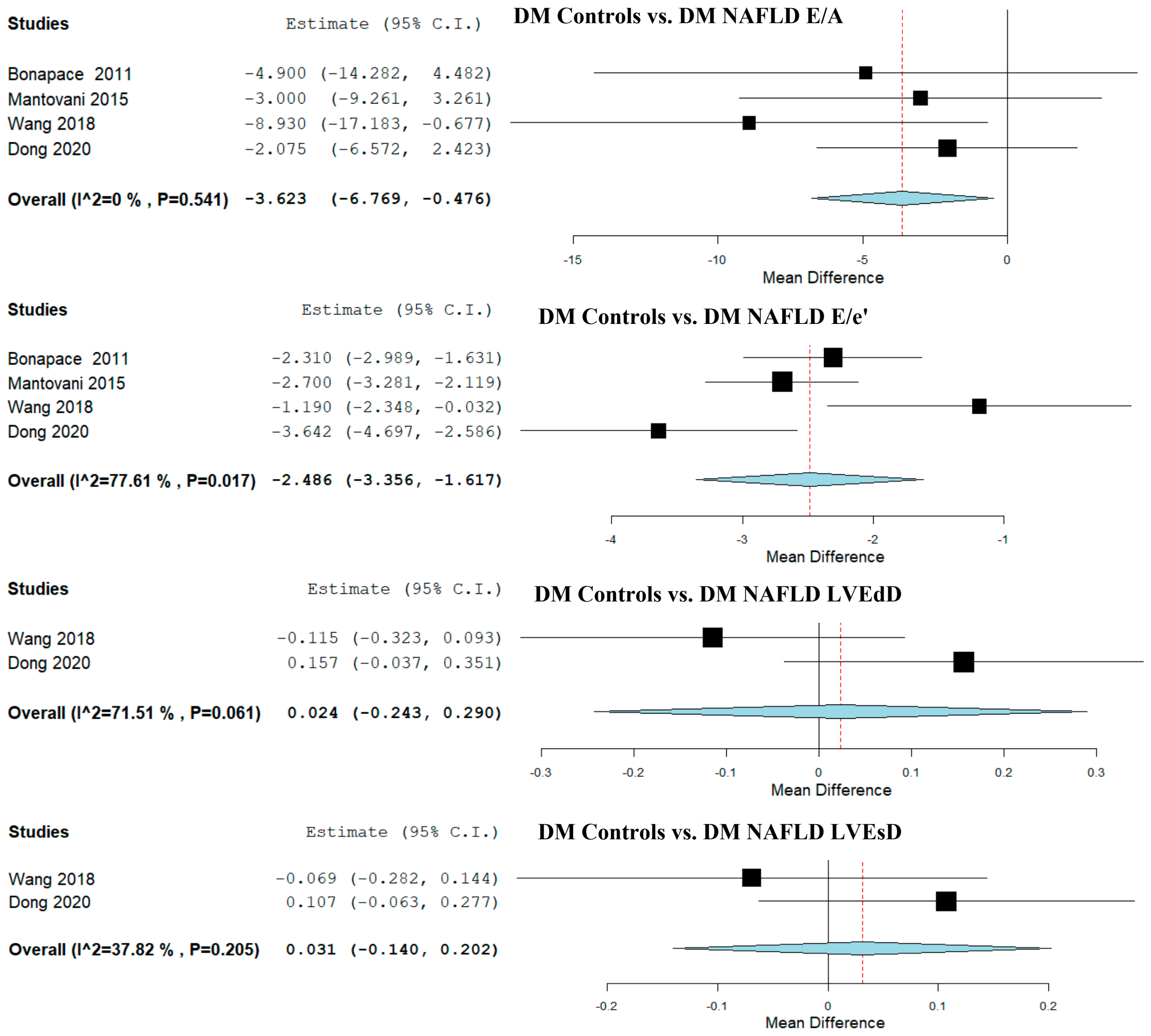

Supplementary Figure S7. Studies evaluating additional echocardiographic parameters including: LVEF, E/A, E/e', LVEdD, LVEsD, E/SRearly, LVMi in DM Controls vs. DM NAFLD.
A- late diastolic filling velocity; E- early diastolic filling velocity; e´- early diastolic mitral annular velocity; SRearly early diastolic strain rate LVEF- Left ventricular ejection fraction; LVEdD- Left ventricular end-diastolic diameter; LVEsD- Left ventricular end-systolic diameter; LVMi- Left ventricular mass index; NAFLD- Non-alcoholic liver disease; SRearly Strain rate early; SRlate- Strain rate late; DM- Diabetes Mellitus.

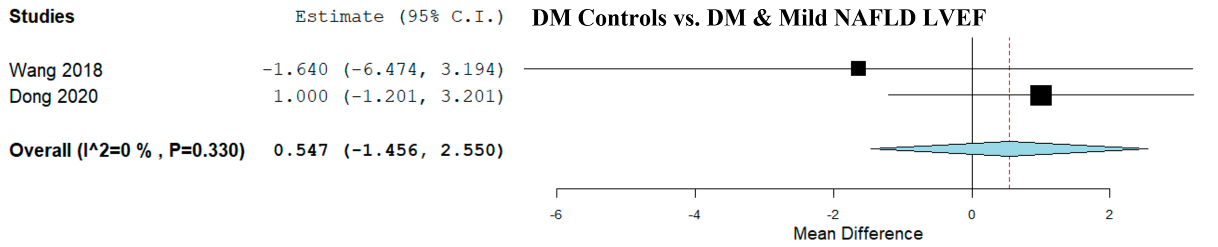

Supplementary Figure S8. Studies evaluating additional echocardiographic parameter including: LVEF in DM Controls vs. DM and mild NAFLD.
LVEF- Left ventricular ejection fraction; NAFLD- Non-alcoholic liver disease, DM- Diabetes Mellitus.

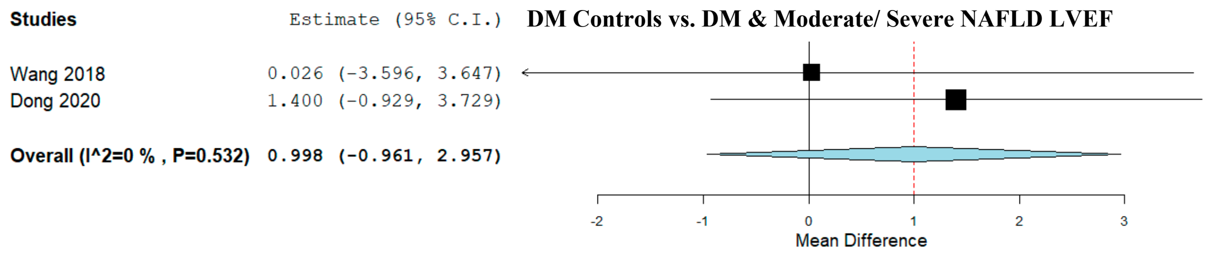

Supplementary Figure S9. Studies evaluating additional echocardiographic parameter including: LVEF in DM Controls vs. DM and Moderate/ Severe NAFLD.
LVEF- Left ventricular ejection fraction; NAFLD- Non-alcoholic liver disease, DM- Diabetes Mellitus.

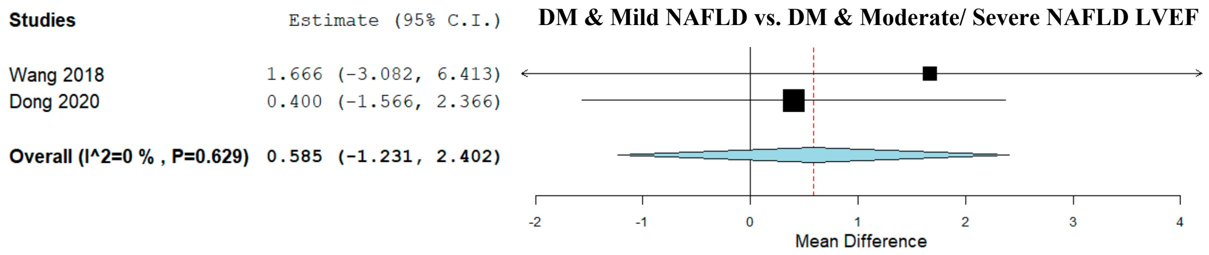

Supplementary Figure S10. Studies evaluating additional echocardiographic parameter including: LVEF in DM and mild NAFLD vs. DM and Moderate/ Severe NAFLD.
LVEF- Left ventricular ejection fraction; NAFLD- Non-alcoholic liver disease, DM- Diabetes Mellitus.

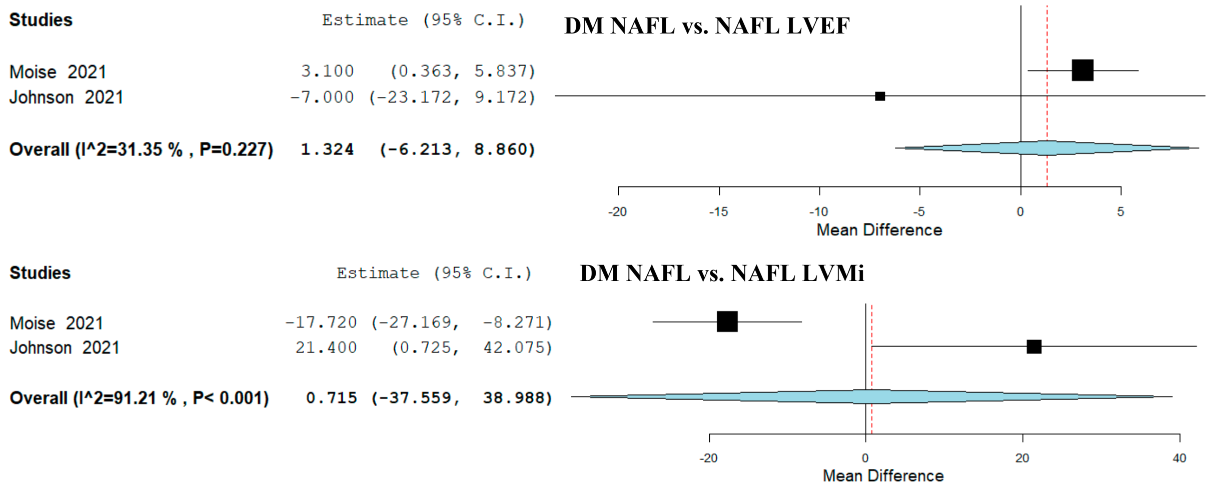

Supplementary Figure S11. Studies evaluating additional echocardiographic parameters including: LVEF and LVMi in DM NAFL vs NAFL.
LVEF- Left ventricular ejection fraction; LVMi- Left ventricular mass index; NAFLD- Non-alcoholic liver disease, DM- Diabetes Mellitus.

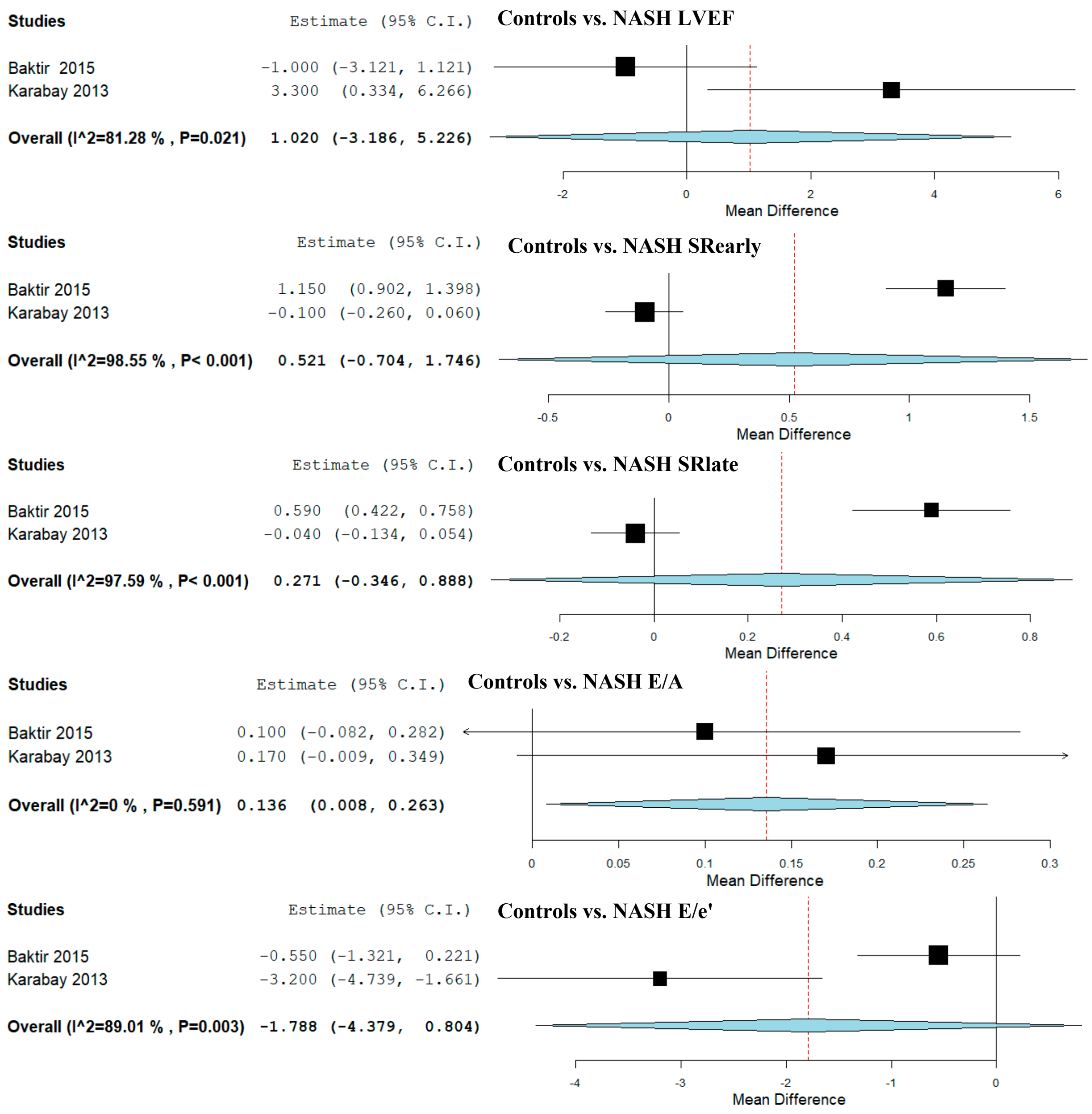

Supplementary Figure S12. Studies evaluating additional echocardiographic parameters including: LVEF, E/A, E/e', SRearly, SRlate in Controls vs. NASH.

A- late diastolic filling velocity; E- early diastolic filling velocity; e´- early diastolic mitral annular velocity; SRearly early diastolic strain rate; SRlate- late diastolic strain rate; LVEF- Left ventricular ejection fraction; NASH- Non-alcoholic steatohepatitis.

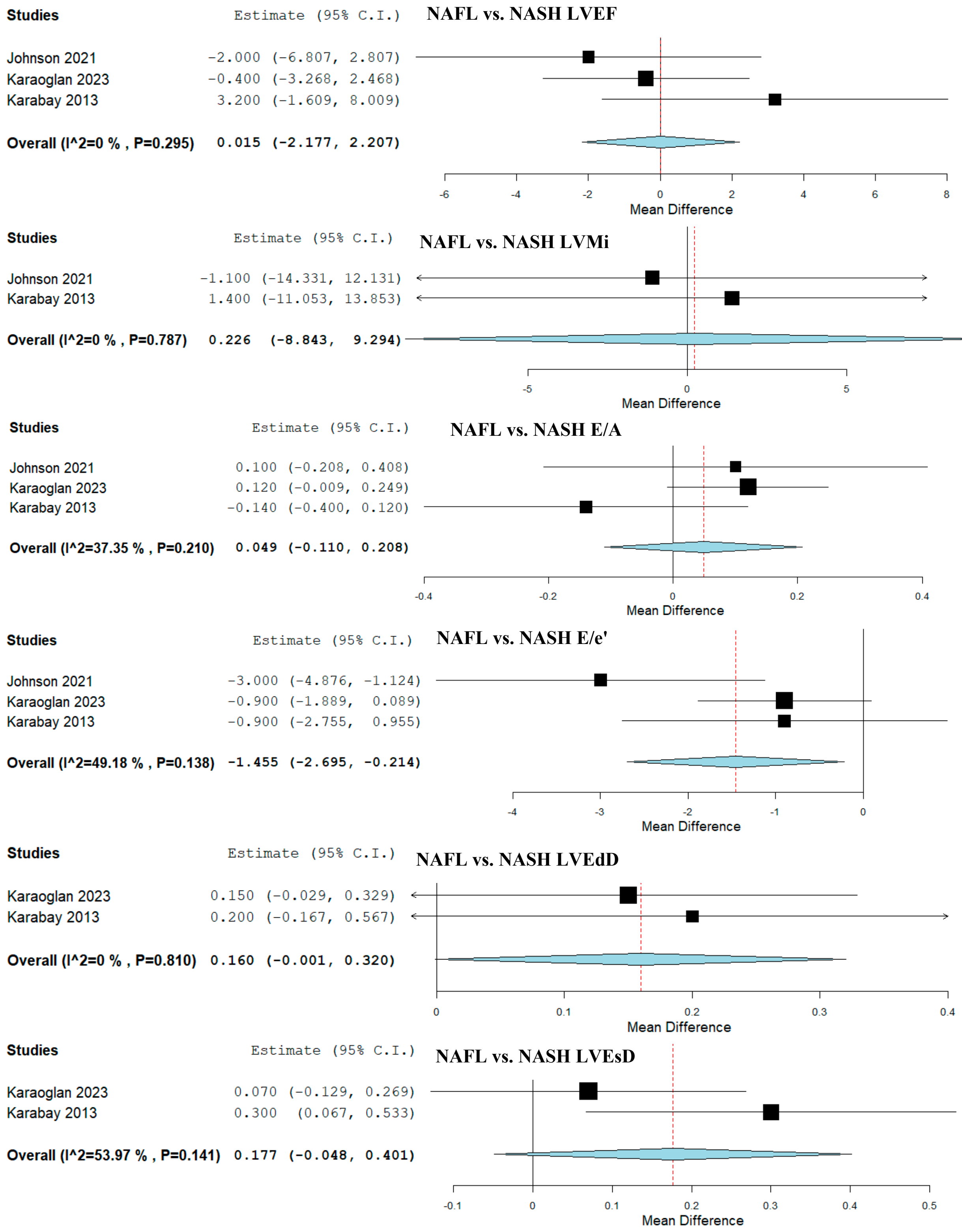

Supplementary Figure S13. Studies evaluating additional echocardiographic parameters including: LVEF, E/A, E/e', LVMi, LVEdD and LVEsD in NAFL vs. NASH.

A- late diastolic filling velocity; E- early diastolic filling velocity; e´- early diastolic mitral annular velocity; LVEF- Left ventricular ejection fraction; LVEdD- Left ventricular end-diastolic diameter; LVEsD- Left ventricular end-systolic diameter; LVMi- Left ventricular mass index; NAFL- Non-alcoholic fatty liver; NASH- Non-alcoholic steatohepatitis.
