# Supplementary material for: When the Liver Echoes to the Heart: Assessing Subclinical Cardiac Dysfunction in NAFLD Using Speckle Tracking Echocardiography—A Systematic Review and Meta-Analysis
Source: Biomedicines. 2025 Nov 27;13(12):2908. doi: 10.3390/biomedicines13122908 (PMC12730947; doi:10.3390/biomedicines13122908)
Supplement: Supplementary file 1 [file biomedicines-13-02908-s001.zip › Supplementary Material S1.pdf]

# Supplementary Material 1

**Search Strategy**

*PubMed:* (("Non-alcoholic Fatty Liver Disease"[Mesh]) OR ("Non-alcoholic Fatty Liver Disease"[All Fields]) OR ("NAFLD") OR ("MAFLD") OR ("metabolic associated fatty liver disease") OR ("metabolic-dysfunction-associated fatty liver disease") OR ("metabolic dysfunction associated fatty liver disease")) AND (("Global Longitudinal Strain"[Mesh]) OR ("Global Longitudinal Strain"[All Fields]) OR ("Ventricular Dysfunction, Left"[Mesh]) OR ("Ventricular Dysfunction, Left"[All Fields]) OR ("speckle tracking") OR ("speckle tracking echocardiography") OR ("subclinical systolic") OR ("subclinical systolic dysfunction"))

*Embase:* ('Non-alcoholic Fatty Liver Disease'/exp OR 'Non-alcoholic Fatty Liver Disease' OR 'NAFLD' OR 'MAFLD' OR 'metabolic associated fatty liver disease' OR 'metabolic-dysfunction-associated fatty liver disease' OR 'metabolic dysfunction associated fatty liver disease') AND ('Global Longitudinal Strain'/exp OR 'Global Longitudinal Strain' OR 'Left Ventricular Dysfunction'/exp OR 'Left Ventricular Dysfunction' OR 'speckle tracking' OR 'speckle tracking echocardiography' OR 'subclinical systolic' OR 'subclinical systolic dysfunction')

*Scopus*: (("Non-alcoholic Fatty Liver Disease"[Mesh]) OR ("Non-alcoholic Fatty Liver Disease"[All Fields]) OR ("NAFLD") OR ("MAFLD") OR ("metabolic associated fatty liver disease") OR ("metabolic-dysfunction-associated fatty liver disease") OR ("metabolic dysfunction associated fatty liver disease")) AND (("Global Longitudinal Strain"[Mesh]) OR ("Global Longitudinal Strain"[All Fields]) OR ("Ventricular Dysfunction, Left"[Mesh]) OR ("Ventricular Dysfunction, Left"[All Fields]) OR ("speckle tracking") OR ("speckle tracking echocardiography") OR ("subclinical systolic") OR ("subclinical systolic dysfunction"))

Extracted data included author names, publication year, country, study design, as well as study characteristics including total subjects, mean age, gender, NAFLD diagnosis, NAFLD percentage, subjects with DM, LVEF, and STE parameters. The final data was collected and presented in the manuscript text.
