# Supplementary material for: When the Liver Echoes to the Heart: Assessing Subclinical Cardiac Dysfunction in NAFLD Using Speckle Tracking Echocardiography—A Systematic Review and Meta-Analysis
Source: Biomedicines. 2025 Nov 27;13(12):2908. doi: 10.3390/biomedicines13122908 (PMC12730947; doi:10.3390/biomedicines13122908)
Supplement: Supplementary file 1 [file biomedicines-13-02908-s001.zip › Supplementary Tables S1 and S2.pdf]

Supplementary Table S1. Studies evaluating STE parameters in NAFLD patients

First Author / Year / Country	Study Design	Study Characteristics	Main Findings
Bonapace S et al. / 2011 / Italy	Cross- sectional	• **Total subjects:** 50 (Controls- 18; NAFLD 32)<br>• **NAFLD:** 64%<br>• **Mean age (years):** Controls 63.0 ± 6; NAFLD 64.8 ± 4<br>• **BMI (kg/m^2):** Controls 28.6 ± 3.5; NAFLD 28.6 ± 3.3<br>• **NAFLD diagnosis:** Ultrasound (US)<br>• **Gender (males):** 38 (76%)<br>• **LV dysfunction assessment:** Echocardiography results:<br>***LVEF (%):*** Controls 71.3 ± 6.9; NAFLD 73.7 ± 7.2<br>***GLS (%):*** Controls -16.1 ± 2.9; NAFLD -15.5 ± 3.0<br>***GSRS (s^{-1}):*** Controls -1.03 ± 0.15; NAFLD -1.01 ± 0.13<br>***SRearly (s^{-1}):*** Controls 0.94 ± 0.20; NAFLD 0.84 ± 0.22<br>***SRlate (s^{-1}):*** Controls 1.0 ± 0.18; NAFLD 0.90 ± 0.21<br>***E/SRE:*** Controls 0.63 ± 0.19; NAFLD 0.77 ± 0.21<br>***LVMi (g/m^2):*** Controls 107.9 ± 14.9; NAFLD 112.8 ± 18.4	NAFLD was associated with reduced GLS and diastolic dysfunction, indicating subclinical myocardial impairment. These changes were detectable despite normal ejection fraction, emphasizing the sensitivity of strain imaging..
Karabay C et al. / 2013 / Turkey	Cross-sectional	• **Total subjects:** 76 (Controls- 21; NAFLD: Simple steatosis- 9; Borderline NASH- 24; Definitive NASH- 22<br>• **NAFLD:** 72%<br>• **Mean age (years):** Controls 40.5 ± 7.8; Simple Steatosis 44.4 ± 4.3; Borderline NASH 42.5 ± 8.5; Definitive NASH 42.8 ± 9.9<br>• **BMI (kg/m^2):** Controls 27.3±3.6; Simple Steatosis 28.3±3.7; Borderline NASH 30.6±3.5; Definitive NASH 32.2±1.4<br>• **NAFLD diagnosis:** Biopsy<br>• **Gender (males):** 43(56,5%)<br>• **LV dysfunction assessment:** Echocardiography results:<br>***LVEF (%):*** Controls 62.5 ± 4.5; Simple Steatosis 62.4 ± 6.5; Borderline NASH 62.2 ± 10.3; Definitive NASH 59.2 ± 5.4<br>***GLS (%):*** Controls –19.8 ± 3.1; Simple Steatosis –17.0 ± 1.2; Borderline NASH –17.1 ± 2.2; Definitive NASH 17.7 ± 2.7<br>***GSRS (s^{-1}):*** Controls – 1.2 ± 0.54; Simple Steatosis – 0.87 ± 1.3; Borderline NASH – 0.92 ± 0.17; Definitive NASH – 1.0 ± 0.22<br>***SRearly (s^{-1}):*** Controls 1.2 ± 0.19; Simple Steatosis 1.2 ± 0.20; Borderline NASH 1.1 ± 0.30; Definitive NASH 1.3 ± 0.33<br>***SRlate (s^{-1}):*** Controls 0.95 ± 0.13; Simple Steatosis 0.91 ± 0.19; Borderline NASH 0.87 ± 0.27; Definitive NASH 0.99 ± 0.18<br>***LVMi (g/m^2):*** Controls 67.6 ± 12.7; Simple Steatosis 81.8 ± 15.7; Borderline NASH83.6 ± 19.6; Definitive NASH 80.4 ± 16.9<br>***LVEdD (cm):*** Controls 4.8 ± 0.4; Simple Steatosis 4.9 ± 0.5; Borderline NASH 4.8 ± 0.3; Definitive NASH 4.7 ± 0.4<br>***LVEsD (cm):*** Controls 3.3 ± 0.2; Simple Steatosis 3.4 ± 0.3; Borderline NASH 3.1 ± 0.2; Definitive NASH 3.1 ± 0.3	GLS impairment was the primary finding in NAFLD patients, suggesting early systolic dysfunction. The preservation of radial and circumferential strains may indicate localized myocardial changes in NAFLD.
Khoshtbaten M et al. / 2015/ Iran	Cross Sectional	• **Total subjects:** 60 (Controls -30; NAFLD-30)<br>• **NAFLD:** 50%<br>• **Mean age (years):** Controls 40.53 ± 8.08 NAFLD 39.79 ± 6.84<br>• **BMI (kg/m^2):** Controls 24.95 ± 1.74; NAFLD 27.33 ± 2.41<br>• **NAFLD diagnosis:** US<br>• **Gender (males):** 36 (60%)<br>• **LV dysfunction assessment:** Echocardiography results:<br>***LVEF (%):*** Controls 57.43 ± 7.3; NAFLD 54.81 ± 6.72<br>***GLS (%):*** Controls -20.27 ± 1.72; NAFLD -18.96 ± 2.31<br>***E/A:*** Controls 1.27 ± 0.18; NAFLD 1.12 ± 0.24<br>***LVM (g):*** Controls 118.8 ± 32.98; NAFLD 162.84 ± 43.06<br>***LVMi (g/m^2):*** Controls 65.23 ± 11.28; NAFLD 83.99 ± 20.83<br>***LVEdD (cm):*** Controls 4.46 ± 0.33; NAFLD 4.61 ± 0.36<br>***LVEsD (cm):*** Controls 30.08 ± 0.3; NAFLD 3.18 ± 0.33	NAFLD patients showed significant impairments in GLS and GRS compared to controls, reflecting early subclinical dysfunction. Circumferential strain preservation indicates selective myocardial layer involvement.
Baktir Aet al. / 2015 / Turkey	Cross- sectional	• **Total subjects:** 56 (Controls -28; NASH-28)<br>• **NASH:** 50%<br>• **Mean age (years):** Controls 41.2±9 NAFLD 41.6±9.8<br>• **BMI (kg/m^2):** Controls 26.7±1.7; NAFLD 27.7±1.6	NASH patients exhibited specific reductions in GLS and GRS, marking early subclinical myocardial dysfunction compared to controls. Circumferential strain was preserved.

		• **NAFLD diagnosis:** Biopsy<br>• **Gender (males):** -<br>• **LV dysfunction assessment:** Echocardiography results:<br>***LVEF (%):*** Controls 65.7±2.4; NAFLD 66.7±5.2<br>***GLS (%):*** Controls -23.73±2.34; NAFLD -18.88±1.51<br>***GSRS (s^{-1}):*** Controls -1.73±-0.28; NAFLD -1.14±0.20<br>***SRearly (s^{-1}):*** Controls 2.35±0.55; NAFLD 1.20±0.38<br>***SRlate (s^{-1}):*** Controls 1.47±0.37; NAFLD 0.88±0.26<br>***E/A:*** Controls 1.23 ± 0.3; NAFLD 1.13 ± 0.39<br>***E/e':*** Controls 6.38 ±1.33; NAFLD 6.93 ± 1.6	
Mantovani A et al. / 2015 / Italy	Cross- sectional	• **Total subjects (All DM):** 222 (Controls- 64; NAFLD- 158)<br>• **NAFLD:** 71%<br>• **Mean age (years):** Controls 66.9 ± 7; NAFLD 68.6 ± 7<br>• **BMI (kg/m^2):** Controls 27.4 ± 3; NAFLD 29.3 ± 5<br>• **NAFLD diagnosis:** US<br>• **Gender (males):**156 (70%)<br>• **LV dysfunction assessment:** Echocardiography results:<br>***LVEF (%):*** Controls 65.4 ± 7; NAFLD 62.8 ± 6<br>***GLS (%):*** Controls -16.2 ± 2.3; NAFLD -15.9 ± 3.0<br>***GSRS (s^{-1}):*** Controls -1.05 ± 0.15; NAFLD -1.02 ± 0.25<br>***SRearly (s^{-1}):*** Controls 1.14 ± 0.26; NAFLD 1.05 ± 0.27<br>***SRlate (s^{-1}):*** Controls 1.08 ± 0.23; NAFLD 1.16 ± 0.36<br>***E/A:*** Controls 0.78 ± 0.2; NAFLD 0.74 ± 0.2<br>***E/e':*** Controls 6.9 ± 2; NAFLD 9.6 ± 2<br>***LVMi (g/m^2):*** Controls 103.7 ± 20; NAFLD 106.7 ± 25	NAFLD independently reduced GLS compared to controls, indicating early subclinical systolic dysfunction. The study highlighted that these changes occur despite normal ejection fraction, underscoring the sensitivity of STE over traditional metrics.
VanWagner L et al. / 2015 / USA	Cross- sectional	• **Total subjects:** 2713 (Controls- 2442; NAFLD- 271)<br>• **NAFLD:** 9,9%<br>• **Mean age (years):** Controls 50.1 ± 3.6; NAFLD 50.5 ± 3.7<br>• **BMI (kg/m^2):** Controls 29.7 ± 6.9; NAFLD 36.2 ± 7.5<br>• **NAFLD diagnosis:** CT<br>• **Gender (males):**1118 (41%)<br>• **LV dysfunction assessment:** Echocardiography results:<br>***LVEF (%):*** Controls 61.6 ± 7.0; NAFLD 62.0 ± 7.7<br>***GLS (%):*** Controls -15.2 ± 2.4; NAFLD -14.2 ± 2.4<br>***GCS (%):*** Controls -15.4 ± 2.8; NAFLD -15.0 ± 3.0<br>***E/A:*** Controls 1.3 ± 0.4; NAFLD 1.2 ± 0.3<br>***E/e':*** Controls 7 ± 2.3; NAFLD 7.7 ± 2.6<br>***LVM (g):*** Controls 164.1 ± 49.1; NAFLD 196.5 ± 57	In conclusion, NAFLD is independently associated with subclinical myocardial remodeling and dysfunction, irrespective of traditional heart failure risk factors, such as obesity, dyslipidemia, hypertension, and diabetes.. NAFLD appears to contribute significantly to the development of HF, particularly HF with HFpEF. The observed relationship between NAFLD and subclinical myocardial dysfunction provides valuable pathophysiological insights into the potential mechanistic link between NAFLD and HF.
Wang Q et al. / 2018 / China	Cross- sectional	• **Total subjects:** 120 (Controls- 40; Diabetes- 40; Diabetes + NAFLD -40)<br>• **NAFLD:** 33%<br>• **Mean age (years):** Controls 61.9±6.9; DM 60.8±8.1; DM + NAFL 64.4±7.9<br>• **BMI (kg/m^2):** Controls 24.74 ± 2.07; DM 24.50 ± 2.58; DM + NAFLD 25.73 ± 2.97<br>• **NAFLD diagnosis:** US<br>• **Gender (males):** 62 (52%)<br>• **LV dysfunction assessment:** Echocardiography results:<br>***LVEF (%):*** Controls 62.52 ± 5.05; DM 61.59 ± 7.06; DM + NAFLD 60.80 ± 7.48<br>***GLS (%):*** Controls -19.86 ± 2.59; DM -17.32 ± 2.43; DM + NFLD -14.28 ± 3.08<br>***GCS (%):*** Controls -18.93 ± 3.13; DM -17.24 ± 2.69; DM + NAFLD -16.00 ± 3.24<br>***GAS (%):*** Controls - 30.98 ± 4.69; DM- 30.22 ± 3.44; DM + NAFLD - 26.67 ± 4.72<br>***GRS (%):*** Controls 53.22 ± 10.00; DM 47.73 ± 7.91; DM + NAFLD 40.92 ± 10.79<br>***E/A:*** Controls 0.85 ± 0.20; DM 0.86± 0.22; DM + NAFLD 0.78 ± 0.23<br>***E/e':*** Controls 7.14 ± 2.15; DM 7.67 ± 2.74; DM + NAFLD 8.86 ± 2.54<br>***LVMi (g/m^2):*** Controls 81.39 ± 17.77; DM 81.81 ± 16.53; DM + NAFLD 90.74 ± 20.88<br>***LVEdD (cm):*** Controls 4.46 ± 0.38; DM 4.53 ± 0.41; DM+ NAFLD 4.648 ± 0.52	NAFLD severity correlated with a stepwise worsening of strain parameters, with the most pronounced reductions observed in patients with severe NAFLD. GLS and GRS showed the highest degree of impairment compared to controls. This highlights the additive impact of NAFLD on myocardial dysfunction in diabetic patients, detectable even in the absence of overt cardiac symptoms.

		LVEsD (cm): Controls 2.79 ± 0.45; DM 2.92 ± 0.43; DM + NAFLD 2.99 ± 0.52<br><br>**Grouping according to NAFLD severity:**<br>***LVEF (%):*** Mild 60.10 ± 7.45; Moderate 58.42 ± 7.00; Severe 58.45 ± 6.36<br>***GLS (%):*** Mild -16.80 ± 2.86; Moderate -14.08 ± 2.43; Severe -12.36 ± 2.94<br>***GCS (%):*** Mild -17.05 ± 3.27; Moderate - 15.83 ± 3.07; Severe - 14.27 ± 3.06<br>***GAS (%):*** Mild - 30.40 ± 4.12; Moderate -26.50 ± 3.45; Severe -23.55 ± 4.99<br>***GRS (%):*** Mild 48.29 ± 9.42; Moderate 40.77 ± 6.49; Severe 35.09 ± 8.37	
Zamirian M et al. / 2018 / Iran	Cross- sectional	• **Total subjects**: 60 (Control-30; NAFLD-30)<br>• **NAFLD:** 50%<br>• **Mean age (years):** Controls 36.9 ± 4.5; NAFLD 38.4 ± 5<br>• **BMI (kg/m^2):** Controls 25.73 ± 2.29; NAFLD 25.84 ± 2.16<br>• **NAFLD diagnosis:** liver biopsy<br>• **Gender (males):** 31 (51%)<br>• **LV dysfunction assessment:** Echocardiography results:<br>***LVEF (%):*** Controls 57.1 ± 5.2; NAFLD 56.7 ± 4.6<br>***GLS (%):*** Controls -21.2 ± 1.4; NAFLD -19.3 ± 2<br>***E/A:*** Controls 1 ± 0.3; NAFLD 0.9 ± 0.3<br>***E/e':*** Controls 7.4 ± 1.2; NAFLD 8.4 ± 0.8<br>***LVEdD (cm):*** Controls 4.52 ± 0.31; NAFLD 4.19 ± 0.17<br>***LVEsD (cm):*** Controls 3.4 ± 1.8; NAFLD 3.21 ± 0.14	NAFLD patients demonstrated subclinical LV systolic and diastolic dysfunction, with significant reductions in GLS compared to controls. The elevated E/e' ratio suggests impaired diastolic relaxation, further highlighting the myocardial impact of NAFLD. The study supports the use of STE as a more sensitive tool for detecting early cardiac changes in these patients.
Dong Y. et al / 2020 / China	Cross-sectional	• **Total subjects**: 97 (All DM) (Control- 30; Mild NAFLD- 32; Moderate-Severe NAFLD- 35)<br>• **NAFLD:** (69%)<br>• **Mean age (years):** Control 48.5 ± 10.0; Mild NAFLD 45.3 ± 5.4; Moderate-Severe NAFLD 47.2 ± 9.7<br>• **BMI (kg/m^2):** Control 24.4 ± 2.6; Mild NAFLD 25.5 ± 3.2; Moderate-Severe 27.3 ± 3.8<br>• **NAFLD diagnosis:** US<br>• **Gender:** (males):69 (71%)<br>• **LV dysfunction assessment:** Echocardiography results:<br>***LVEF (%):*** Controls 60.3 ± 5.0; Mild NAFLD 59.3 ± 3.7; Moderate-Severe NAFLD 58.9 ± 4.5<br>***GLS (%):*** Controls -19.0 ± 2.6; Mild NAFLD -17.9 ± 3.1; Moderate-Severe NAFLD -14.1 ± 4.1<br>***GRS (%):*** Controls 45.7 ± 6.2; Mild NAFLD 45.1 ± 9.0; Moderate-Severe NAFLD 40.8 ± 6.8<br>***GCS (%):*** Controls -19.5 ± 3.3; Mild NAFLD -19.4 ± 2.6; Moderate-Severe NAFLD -17.2 ± 3.5<br>***GAS (%):*** Controls -29.0 ± 3.4; Mild NAFLD -27.2 ± 4.3; Moderate-Severe NAFLD -24.3 ± 4.7<br>***E/A:*** Controls 1.15 ± 0.36; Mild NAFLD 1.12 ± 0.31; Moderate-Severe NAFLD 1.10 ± 0.32<br>***E/e':*** Controls 7.6 ± 2.0; Mild NAFLD9.1 ± 2.3; Moderate-Severe NAFLD 13.2 ± 2.7<br>***LVMi (g/m^2):*** Controls 68.9 ± 11.4; Mild NAFLD 70.4 ± 8.6, Moderate-Severe NAFLD 71.5 ± 7.3<br>***LVEdD (cm):*** Controls 4.75 ± 0.45; Mild NAFLD 4.4 ± 0.23; Moderate-Severe NAFLD 4.77 ± 0.53<br>***LVEsD (cm):*** Controls 3.07 ± 0.39; Mild NAFLD 2.78 ± 0.23; Moderate-Severe 3.13 ± 0.46	Patients with moderate-to-severe NAFLD showed progressive impairment in all strain parameters compared to milder forms, highlighting subclinical LV dysfunction. These changes were independent of BMI, suggesting a direct relationship with liver disease severity.
Chiu L et al. / 2020 / USA	Cross- sectional	• **Total subjects:** 2356 (Control- 1972; NAFLD- 384)<br>• **NAFLD:**16%<br>• **Mean age (years):** Controls 52 ± 12; NAFLD 53 ± 12<br>• **BMI (kg/m^2):** Controls 26.8 ± 4.9; NAFLD 31.6 ± 6.0<br>• **NAFLD diagnosis:** CT<br>• **Gender (males):**1133 (48%)<br>• **LV dysfunction assessment:** Echocardiography results:<br>***EF:*** Controls 66 ± 6; NAFLD 66 ± 5<br>***GLS (%):*** Controls -20 ± 3; NAFLD -19 ± 3<br>***E/A:*** Controls 1.17 ± 0.36; NAFLD 1.05 ± 0.25<br>***E/e':*** Controls 6 ± 1.48; NAFLD 7 ± 1.48<br>***LVMi (g/m^2):*** Controls 85 ± 18; NAFLD 87 ± 17<br>***LVEdD (cm):*** Controls 4.9 ± 0.4; NAFLD 4.9 ± 0.4<br>***LVEsD (cm):*** Controls 3.1 ± 0.4; NAFLD 3.1 ± 0.4	Diabetes exacerbates GLS reductions in NAFLD patients compared to those without diabetes. The findings demonstrate that diabetes and NAFLD synergistically impair myocardial function, specifically in the longitudinal direction.
Johnson P / 2021 / USA	Cross- sectional	• **Total subjects** 33 (NAFL 17 (3 + DM); NASH 16 (9 + DM))<br>• **NAFL**: 51%	NAFLD patients exhibited reduced GLS and impaired diastolic function, highlighting

		• **Mean age (years):** NAFL 46.3 ± 10.1; NASH 51.4 ± 11.2<br>• **BMI (kg/m^2):** NAFL 36.4 ± 8.2; NASH 35.3 ± 6.6<br>• **NAFL diagnosis:** Liver Biopsy<br>• **Gender (males):** 20 (60%)<br>• **LV dysfunction assessment:** Echocardiography results:<br>***NAFL vs. NASH***<br>***LVEF (%):*** NAFL 62.0 ± 8.0; NASH 64.0 ± 6.0<br>***GLS (%):*** NAFL -17.2 ± 2.6; NASH -18.3 ± 3.5<br>***E/A:*** NAFL 1.2 ± 0.5; NASH 1.1 ± 0.4<br>***E/e':*** NAFL 8.8 ± 2; NASH 11.8 ± 3.3<br>***LVMi (g/m^2):*** NAFL 81.2 ± 18.7; NASH 82.3 ± 20<br><br>***NAFL vs. NASH (WITHOUT DM)***<br>***LVEF (%):*** NAFL 63.3 ± 6.2; NASH 65.3 ± 6.2<br>***GLS (%):*** NAFL -17.1 ± 2.5; NASH -19.4 ± 3.8<br>***E/A:*** NAFL1.3 ± 0.5; NASH 1.2 ± 0.9<br>***E/e':*** NAFL 8.6 ± 2; NASH 11.8 ± 3.1<br>***LVMi (g/m^2):*** NAFL 77.4 ± 17.4; NASH 79.1 ± 18.8<br><br>***NAFL vs. NASH (WITH DM)***<br>***LVEF (%):*** NAFL 56.3 ± 14.0; NASH 63.6 ± 6.8<br>***GLS (%):*** NAFL -18.0 ± 3.5; NASH -17.5 ± 3.3<br>***E/A:*** NAFL1.1 ± 0.4; NASH 1 ± 0.3<br>***E/e':*** NAFL 9.7 ± 2.6; NASH 11.8 ± 3.5<br>***LVMi (g/m^2):*** NAFL 98.8 ± 16.4; NASH 84.7 ± 21.6	subclinical myocardial involvement. These changes occurred independently of BMI, underscoring the role of liver-related metabolic disturbances. The study emphasizes the need for cardiac monitoring in NAFLD patients, regardless of obesity.
Moise C et al. / 2021<br>/ Romania	Cross-sectional	• **Total subjects:** 159 (Controls- 80; NAFLD- 35; NAFLD + DM- 44)<br>• **NAFLD:** 49%<br>• **Mean age (years):** Controls 29 ± 5; NAFLD 38 ± 5; NAFLD+DM 31 ± 8<br>• **BMI (kg/m^2):** Controls 23 ± 3.9; NAFLD 30 ± 3; NAFLD + DMT1 27 ± 6<br>• **NAFLD diagnosis:** US<br>• **Gender (males):** 97 (61%)<br>• **LV dysfunction assessment:** Echocardiography results:<br>***LVEF (%):*** Controls 58.2 ± 4.4; NAFLD 57.2 ± 5.2; NAFLD+ DM 60.3 ± 7.2<br>***GLS (%):*** Controls -19.2 ± 2.2 NAFLD -17.7 ± 2; NAFLD+ DM -18 ± 2.2<br>***LVMi (g/m^2):*** Controls 67.04 ±13.8; NAFLD 94.52 ± 19.15; NAFLD +DM 76.8 ± 23.7<br>***LVEdD (cm):*** Controls 45.9 ± 4.7; NAFLD 49.1 ± 5.1; NAFLD + DM 44.5 ± 4.6<br>***LVEsD (cm):*** Controls 30.7 ± 4.4; NAFLD 37.5 ± 4.5; NAFLD + DM 30 ± 4.7	Young adults with NAFLD showed selective GLS reductions in the endocardial layer compared to controls, indicating early subclinical myocardial changes. This localized dysfunction reflects the vulnerability of subendocardial fibers to metabolic disturbances.
Sewan H et al. / 2021 / Iraq	Cross- sectional	• **Total subjects:** 60 (Controls- 30; NAFLD- 30)<br>• **NAFLD:** 50%<br>• **Mean age (years):** Controls 44.6 ± 9.87; NAFLD 47.2 ± 11.13<br>• **BMI (kg/m^2):** Controls 25.38 ± 1.53; NAFLD 28.76 ± 4.91<br>• **NAFLD diagnosis:** US<br>• **Gender (males):**19 (31.6%)<br>• **LV dysfunction assessment:** Echocardiography results:<br>***LVEF (%):*** Controls 65.26 ±2.39; NAFLD 64.93 ± 1.96<br>***GLS (%):*** Controls -23.05 ± 1.19; NAFLD -18.85 ± 1.07<br>***E/A:*** Controls 1.46 ± 0.33; NAFLD 1.27 ± 0.78<br>***E/e':*** Controls 6.85 ± 1.23; NAFLD 9.86 ± 1.69	NAFLD patients exhibited significant impairments in GLS compared to controls, reflecting subclinical systolic dysfunction. Diastolic function was also impaired, as evidenced by a higher E/e' ratio and lower E/A ratio in the NAFLD group. These findings underscore the sensitivity of GLS detecting subclinical LV dysfunction in NAFLD patients, even with preserved ejection fraction.
Ismaiel A et al / 2022 / Romania	Cross-Sectional	• **Total subjects:** 75 (Controls- 37; MAFLD 38)<br>• **MAFLD:** 51%<br>• **Mean age (years):** Controls 32 ± 10; MAFLD 54 ± 8<br>• **BMI (kg/m^2):** Controls 22.3 ± 3.6; MAFLD 30.6 ± 4<br>• **NAFLD diagnosis:** US<br>• **Gender (males):** 35 (47%)	Patients with MAFLD are at an elevated risk of developing both cardiac systolic and subclinical systolic dysfunction, as well as diastolic dysfunction. NAFLD severity, especially in advanced fibrosis stages, correlated with worsening GLS and GRS compared to earlier

		• **LV dysfunction assessment:** Echocardiography results:<br>***LVEF (%):*** Controls 52,78 ± 6.638; MAFLD 49.63 ± 6.85<br>***GLS (%):*** Controls -20.69 ± 2.45; MAFLD -17.65 ± 1.91<br>***E/A:*** Controls 1.72 ± 0.49; MAFLD 1.09 ± 0.55<br>***E/e':*** Controls 4.95 ± 1.27; MAFLD 6.16 ± 1.88	stages. Showing the progression of subclinical myocardial dysfunction with increasing liver disease severity.
Lai Y et al. / 2022 / Taiwan	Cross-Sectional	• **Total subjects:** 2161 (Controls- 1019; NAFLD low fibrosis score-840; NAFLD high fibrosis score-302)<br>• **NAFLD:** 52%<br>• **Mean age (years):** Controls 46 ± 10; NAFLD Low Fibrosis Score 48 ± 9; NAFLD High Fibrosis Score 56 ± 8<br>• **BMI (kg/m^2):** Controls 22.3 ± 2.5; NAFLD, Low Fibrosis Score 25.7 ± 3.2; NAFLD High Fibrosis Score 27.2 ± 3.5<br>• **NAFLD diagnosis:** US<br>• **Gender (males):** 1372 (63%)<br>• **LV dysfunction assessment:** Echocardiography results:<br>***LVEF (%):*** Controls 62.79 ± 5.05; NAFLD 62.21 ± 5.12<br>***GLS (%):*** Controls -20.85 ± 1.91; NAFLD -19.72 ± 1.63<br>***E/A:*** Controls 1.4 ± 0.49; NAFLD 1.15 ± 0.37<br>***E/e':*** Controls 6.71 ± 2.2; NAFLD 7.37 ± 2.64<br>***LVM (g):*** Controls 129.66 ± 29.83; NAFLD 149.9 ± 29.1<br>***LVMi (g/m^2):*** Controls 72.32 ± 13.73; NAFLD 76.35 ± 13.12	NAFLD has been implicated as a contributing factor in the development HFpEF and AF, potentially mediated through the accumulation of epicardial fat. In a large Asian cohort, the findings revealed that hepatic fibrosis in NAFLD is independently associated with left ventricular (LV) diastolic dysfunction, impaired left atrial (LA) deformation, and increased LA stiffness.
Karaoğlan B / 2023 / Turkey	Cross-sectional	• **Total subjects:** 61 (NAFL- 31; NASH- 30)<br>• **NAFL:** 51%<br>• **Mean age (years):** NAFL 54.4 ± 7.8; NASH 51.7 ± 8.4<br>• **BMI (kg/m^2):** NAFL 31 ± 4.8; NASH 30.4 ± 4.0<br>• **NAFLD diagnosis:** Liver Biopsy<br>• **Gender (males):** 21 (34%)<br>• **LV dysfunction assessment:** Echocardiography results:<br>***LVEF (%):*** NAFL 56.8 ± 7; NASH 57.2 ± 4.1<br>***GLS (%):*** NAFL −13.5 ± 4.1; NASH −15.1 ± 2.7<br>***GCS (%):*** NAFL −13.5 ± 3.0; NASH −14.4 ± 2.9<br>***GRS (%):*** NAFL 36.9 ± 10.1; NASH 40.2 ± 8.5<br>***E/A:*** NAFL 0.98 ± 0.29; NASH 0.86 ±0.22<br>***E/e':*** NAFL 7.6 ± 1.2; NASH 8.5 ± 2.5<br>***LVEdD (cm):*** NAFL 49.5 ± 2.9; NASH 48 ± 4.1<br>***LVEsD (cm):*** NAFL 29.6 ± 3.8; NASH 28.9 ± 4.1	NAFLD amplified GLS impairments in metabolic syndrome patients compared to those without NAFLD. This highlights the synergistic impact of metabolic syndrome and NAFLD on myocardial dysfunction.

A—late diastolic filling velocity; BMI—Body mass index; E—early diastolic filling velocity; GAS—Global apical strain; GCS—Global circumferential strain; GLS—Global longitudinal strain; GRS—Global radial strain; GSRS—Global systolic radial strain; HFpEF—Heart failure with preserved ejection fraction; LVEF—Left ventricular ejection fraction; LVEdD—Left ventricular end-diastolic diameter; LVEsD—Left ventricular end-systolic diameter; LVM—Left ventricular mass; LVMi—Left ventricular mass index; NAFL—Non-alcoholic fatty liver; NAFLD—Non-alcoholic liver disease; NASH—Non-alcoholic steatohepatitis; SRearly—Strain rate early; SRlate—Strain rate late; US—Ultrasound

Supplementary Table S2. Newcastle-Ottawa Scale Quality Assessment of Cross-sectional Studies.

Study	Selection (Maximum 5 stars)				Comparability (Maximum 2 stars)	Outcome (Maximum 3 stars)		Score (Total maximum 10 stars)
	Sample Representativeness	Sample-size	Non-Respondents	Ascertainment of the exposure (risk factor)	Comparability	Assessment of the outcome	Statistical Test	
Bonapace et al. 2011	*	-	-	**	*	**	*	7
Karabay et al. 2013	-	-	-	**	* *	**	*	7
Baktir et al. 2015	-	-	-	**	**	**	*	7
Khoshbaten et al. 2015	-	-	-	**	**	**	*	7
Mantovani et al. 2015	*	-	-	**	*	**	*	7
VanWagner et al. 2015	*	-	-	**	*	**	*	7
Wang et al. 2018	-	-	-	**	**	**	*	7
Zamirian et al. 2018	*	-	-	**	**	**	*	8
Dong et al. 2020	-	-	-	**	* *	**	*	7
Chiu et al. 2020	-	-	-	**	*	**	*	6
Johnson et al. 2021	-	-	-	**	**	**	*	7
Moise et al. 2021	-	-	-	**	-	**	*	5
Sewan et al. 2021	*	-	-	**	*	**	*	7
Ismaiel et al. 2022	*	-	-	**	*	**	*	6
Lai et al. 2022	-	-	-	**	*	**	*	6
Karaoglan 2023	*	-	-	**	* *	**	*	8
